# Antidepressant Effects of Lactoferrin-Modified Sodium Alginate-Based Saikosaponin A Nanogels via Intranasal Administration: Involvement of Olfactomedin-Family Proteins

**DOI:** 10.3390/ph19091425

**Published:** 2026-09-09

**Authors:** Jinbang Li, Meng Han, Lihua Cao, Shuo Tian, Mengyun Liu, Hongwei Li, Kai Li, Mingsan Miao, Jianguang Zhu, Le Kang

**Affiliations:** 1 National International Cooperation Base of Chinese Medicine, Henan University of Chinese Medicine, Zhengzhou 450046, China; 2 Collaborative Innovation Center of Research and Development on the Whole Industry Chain of Yu-Yao, Zhengzhou 450046, China

**Keywords:** sodium alginate, lactoferrin, saikosaponin A, nanogel, intranasal delivery, depression, olfactomedin family proteins

## Abstract

**Objective:** To develop a lactoferrin-modified sodium alginate nanogel loaded with saikosaponin A (SA-Lf-NG@SAA) and evaluate its potential antidepressant-like effects following intranasal administration. **Methods:** SA-Lf-NG@SAA nanogels were prepared and optimized using single-factor experiments combined with response surface methodology. The optimized formulation was characterized in terms of particle size, zeta potential, stability, morphology, drug-loading performance, and in vitro release behavior. Male C57BL/6J mice were used to establish a chronic unpredictable mild stress (CUMS) model to evaluate the antidepressant-like effects of SA-Lf-NG@SAA after intranasal administration. Histopathological changes in the hippocampus and striatum were observed by hematoxylin and eosin staining. Western blotting was used to detect the expression of OLFM-1, OLFM-3, and OLFM-4 in olfactory bulb tissues, and immunofluorescence staining was performed to further examine their expression in the hippocampus and striatum. **Results:** The optimized SA-Lf-NG@SAA nanogels had an average particle size of approximately 85 nm and a zeta potential ranging from 0 mV to −10 mV, and they maintained their basic particle characteristics during a 14-day short-term physical stability study after dispersion in PBS. In vitro release analysis showed that SA-Lf-NG@SAA exhibited a distinct initial lag phase, followed by sustained drug release, with a cumulative release rate of approximately 75% at 72 h. Kinetic fitting using zero-order, first-order, Higuchi, and Korsmeyer–Peppas models showed that the post-lag release profile was best described by the Higuchi model, suggesting a prolonged release pattern under the present in vitro conditions. In CUMS mice, intranasal administration of SA-Lf-NG@SAA effectively improved depression-like behaviors, alleviated pathological damage in the hippocampus and striatum, and enhanced neuronal morphology. Western blotting showed that SA-Lf-NG@SAA increased the expression of OLFM-1, OLFM-3, and OLFM-4 in olfactory bulb tissues. Immunofluorescence staining further confirmed that the expression of these proteins was significantly enhanced in the hippocampus and striatum, especially in the high-dose SA-Lf-NG@SAA group. **Conclusions:** SA-Lf-NG@SAA nanogels were successfully developed as a potential nanogel-based formulation intranasal administration of saikosaponin A, showing favorable physicochemical properties, short-term stability, and prolonged release behavior. Intranasal administration of SA-Lf-NG@SAA alleviated depression-like behaviors in CUMS mice and improved histopathological alterations in the hippocampus and striatum. The antidepressant-like effects of SA-Lf-NG@SAA may be associated with the regulation of OLFM-family protein expression in depression-related brain regions. These findings suggest that SA-Lf-NG@SAA has potential as a nanogel-based intranasal formulation for the delivery of active components from traditional Chinese medicine in depression therapy. However, the specific contribution of lactoferrin modification requires further validation using unmodified SA-NG@SAA as a control.

## 1. Introduction

Depression is a common and disabling psychiatric disorder characterized by persistent low mood, anhedonia, cognitive impairment, and recurrent episodes. Although several classes of antidepressants are currently available, their clinical effectiveness is frequently limited by delayed onset of action, inadequate therapeutic response, adverse effects, and poor treatment adherence. These limitations have prompted continued interest in naturally derived compounds with potential antidepressant activity and in drug-delivery systems that may improve their pharmaceutical and therapeutic performance [1].

Saikosaponin A (SAA), a major triterpenoid saponin derived from Bupleurum species, has demonstrated antidepressant-like, anti-inflammatory, and neuroprotective activities in preclinical studies [2,3,4]. However, the pharmaceutical application of SAA remains challenging because of its limited aqueous solubility, suboptimal oral exposure, and potential hemolytic toxicity when administered by injection [5,6,7]. The development of an appropriate formulation and administration route may therefore help improve the delivery of SAA while reducing its dependence on conventional systemic administration.

Intranasal administration has attracted increasing attention as a non-invasive approach for delivering therapeutic agents intended for central nervous system disorders. Drugs administered through the nasal cavity may reach the systemic circulation or gain access to the central nervous system through pathways associated with the olfactory and trigeminal nerves, while avoiding gastrointestinal degradation and first-pass hepatic metabolism [8,9,10]. Nevertheless, rapid mucociliary clearance, limited administration volume, enzymatic degradation, and insufficient residence time can restrict the efficiency of intranasal delivery. Nanogels are hydrated polymeric networks with nanoscale dimensions that can encapsulate bioactive compounds, improve their dispersion, prolong local residence, and provide controlled drug release [11]. These characteristics make nanogels potentially suitable carriers for the intranasal administration of poorly water-soluble natural products.

From the perspective of traditional Chinese medicine, the nose and the brain are considered functionally interconnected. In traditional Chinese medicine, the concept that “the nose connects with the brain” has been used to describe the close relationship between the nasal orifice and brain function. From a modern biomedical perspective, anatomical connections between the nasal cavity and the central nervous system, particularly through the olfactory and trigeminal pathways, provide a physiological basis for investigating intranasal drug delivery to the brain [12]. In traditional Chinese medicine, the brain is regarded as the “sea of marrow” and the residence of mental activity, whereas the nose is closely associated with the circulation of qi and the functional communication between the internal organs and the external environment. Accordingly, intranasal administration has traditionally been used to treat disorders involving consciousness, emotion, cognition, and the head [13,14]. This theoretical understanding provides a traditional medical rationale for exploring intranasal administration in the treatment of depression. However, the traditional concept should be regarded as a theoretical framework that complements, rather than replaces, modern anatomical, pharmacokinetic, and neurobiological evidence.

Sodium alginate is a biocompatible and biodegradable anionic polysaccharide containing abundant carboxyl groups that can participate in ionic gelation and chemical modification. It has therefore been widely investigated as a matrix material for controlled drug-delivery systems [15,16]. Lactoferrin is a naturally occurring iron-binding glycoprotein with favorable biocompatibility and multiple biological activities. Lactoferrin and lactoferrin-modified nanocarriers have been investigated in various drug-delivery applications because of their potential interactions with cells and their possible relevance to the delivery of therapeutic agents to neural tissues [17,18,19]. Accordingly, conjugating lactoferrin to sodium alginate was explored as a strategy for preparing SAA-loaded nanogels for intranasal administration.

The olfactomedin family comprises secreted or membrane-associated proteins involved in diverse biological processes, including neural development, axonal growth, synaptic regulation, immune responses, and cellular survival. OLFM1 has been associated with neuronal development and axonal regulation, whereas OLFM3 has been implicated in central nervous system activity and inflammatory regulation [20,21]. OLFM4 is primarily recognized as an immune-related protein and has also been proposed as a potential biomarker associated with major depressive disorder [22].

In the present study, lactoferrin-modified sodium alginate nanogels loaded with SAA (SA-Lf-NG@SAA) were developed for intranasal administration. The formulation was optimized using single-factor experiments and response surface methodology and was evaluated in terms of particle size, polydispersity index, zeta potential, morphology, stability, drug-loading capacity, encapsulation efficiency, and in vitro release behavior. Its antidepressant-like effects were subsequently investigated in mice exposed to chronic unpredictable mild stress through behavioral testing and histopathological examination of the hippocampus and striatum. In addition, the expression of OLFM1, OLFM3, and OLFM4 was examined in the olfactory bulb, hippocampus, and striatum to explore whether changes in these proteins were associated with the effects of SA-Lf-NG@SAA. The study was designed to provide preliminary evidence for the feasibility of SA-Lf-NG@SAA as an intranasal formulation.

## 2. Results

**Figure 1:**
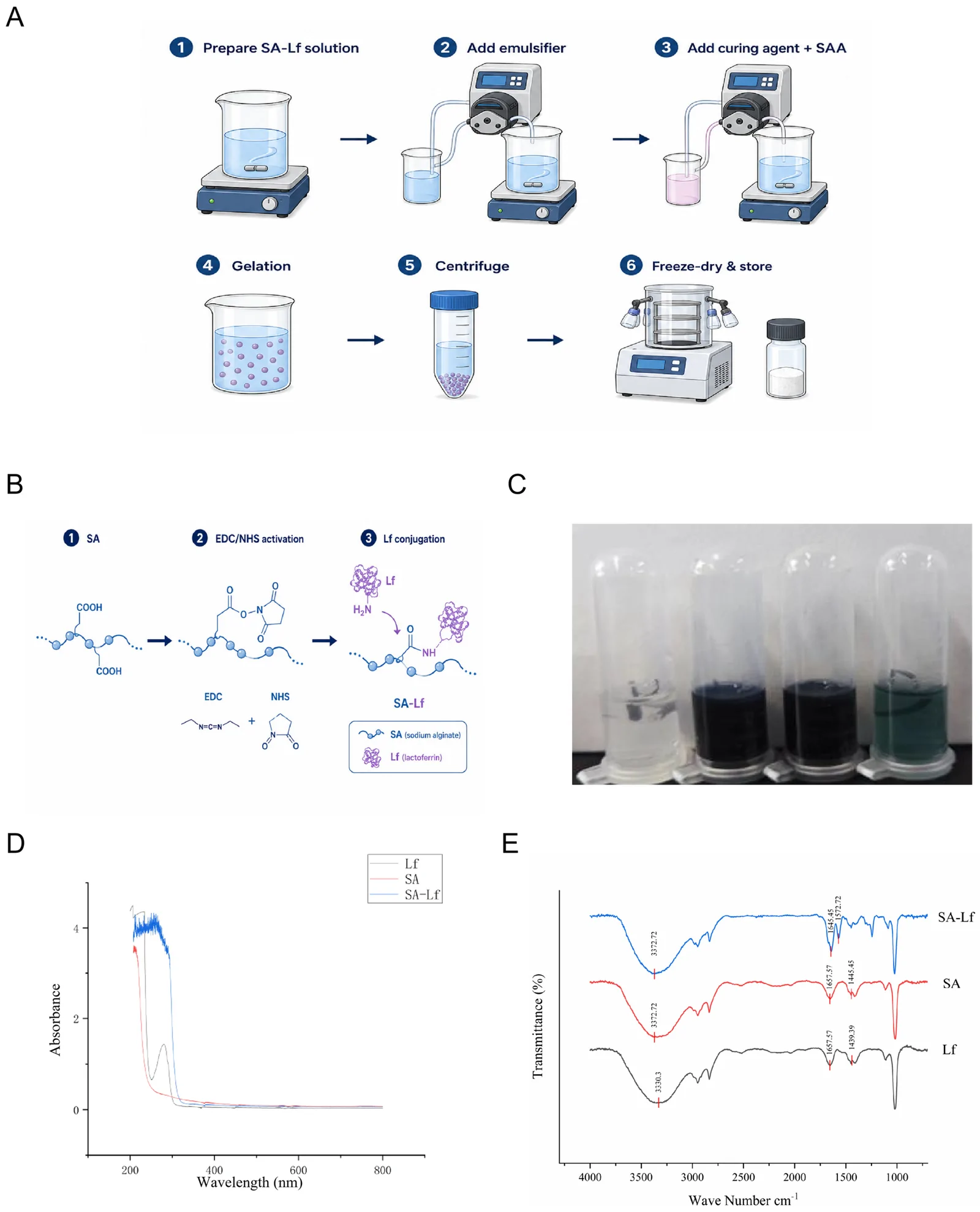
Schematic illustration of the preparation process and characterization of SA-Lf conjugate. (**A**) Schematic representation of the synthesis of SA-Lf conjugate and subsequent fabrication of SA-Lf-NG@SAA. (**B**–**E**) Characterization of SA-Lf conjugate confirming the successful conjugation between SA and Lf. The physicochemical properties of the final SA-Lf-NG@SAA formulation are presented in subsequent figures. (**B**) Schematic illustration of the EDC/NHS-mediated amidation reaction between SA and Lf. (**C**) Chromogenic Reaction of Genipin. (**D**) UV Spectra of SA-Lf, Lf, and SA. (**E**) FTIR spectra of SA, Lf, and SA-Lf.

To clearly present the formulation design strategy, the preparation procedure of SA-Lf-NG@SAA nanogels is schematically illustrated in Figure 1A. Briefly, SA was first conjugated with Lf to obtain the SA-Lf polymer, which was then used as the nanogel matrix. During the emulsification process, the SA-Lf solution was dispersed in the oil phase to form droplets, followed by ionic crosslinking with Zn^2+^ in the presence of SAA. After gelation, centrifugation, washing, and freeze-drying, SAA-loaded lactoferrin-modified sodium alginate nanogels were obtained.

The proposed conjugation mechanism between SA and Lf is shown in Figure 1B. The carboxyl groups of SA were activated by EDC/NHS to form reactive ester intermediates, which subsequently reacted with the amino groups of Lf to generate stable amide bonds. This EDC/NHS-mediated amidation reaction provided the chemical basis for the successful construction of the SA-Lf conjugate.

### 2.1. Confirmation and Characterization of SA-Lf Conjugation

Since SA-Lf conjugation serves as the structural basis for the subsequent preparation of SA-Lf-NG@SAA, FTIR, BCA assay, and UV-visible spectroscopy were initially performed to confirm successful conjugation between SA and Lf.

#### 2.1.1. Qualitative Confirmation of Lf by the Genipin Assay

The genipin chromogenic assay was used to qualitatively examine the distribution of Lf after ultrafiltration. No visible color development was observed in the blank control. In contrast, blue coloration was observed in the Lf-positive control and in both the retained and filtrate fractions obtained after ultrafiltration. The retained fraction exhibited marked blue coloration, confirming the presence of proteinaceous components in the high-molecular-weight fraction. The coloration detected in the filtrate suggested that a proportion of unreacted or unconjugated Lf remained and was removed during ultrafiltration (Figure 1C).

#### 2.1.2. Lf Grafting Ratio Quantification of the Lf Grafting Ratio

A linear relationship was obtained between protein concentration and absorbance in the BCA assay, with the regression equation:

Y = 1373.6X − 191.8(1)

and a coefficient of determination of *R*^2^ = 0.9935. The mean Lf concentration measured in the SA-Lf samples was 356.82 μg/mL, corresponding to an estimated Lf grafting ratio of 35.68% (Supplementary Figure S1 and Table S1).

#### 2.1.3. UV–Visible Spectral Characteristics of SA-Lf

Lf exhibited a characteristic absorption peak at approximately 281 nm, whereas SA showed no distinct absorption peak within the examined wavelength range. The SA-Lf spectrum displayed an absorption peak at approximately 275 nm. The presence of the protein-associated absorption band in the SA-Lf spectrum was consistent with the incorporation of Lf into the SA-containing product (Figure 1D).

#### 2.1.4. FTIR Spectroscopic Characterization

FTIR spectroscopy was further used to characterize the structural changes after the conjugation of SA with Lf. As shown in Figure 1E, Lf exhibited a broad absorption band at 3330.3 cm^−1^, which was attributed to O–H and N–H stretching vibrations. A characteristic absorption band was also observed at 1657.57 cm^−1^, corresponding mainly to the amide I band of protein. SA showed a broad O–H stretching band at 3372.72 cm^−1^, together with absorption bands at 1657.57 and 1445.45 cm^−1^, which were associated with carboxylate-related vibrations of alginate.

Compared with SA and Lf, the FTIR spectrum of SA-Lf showed obvious changes in the amide-related region. In particular, the absorption band shifted to 1645.45 cm^−1^, and an additional band appeared at 1572.72 cm^−1^. The band at approximately 1645 cm^−1^ can be assigned to amide I-related C=O stretching, whereas the band at approximately 1573 cm^−1^ is consistent with amide II-related N–H bending and C–N stretching vibrations. These spectral changes indicate the formation of amide-related structures after EDC/NHS-mediated reaction between the carboxyl groups of SA and the amino groups of Lf. Together with the genipin chromogenic assay, BCA analysis, and UV-visible spectroscopy results, the FTIR results further support the successful conjugation of Lf with SA.

### 2.2. Formulation Optimization Results for SA-Lf-NG@SAA

#### 2.2.1. Effects of Individual Formulation Variables

**Figure 2:**
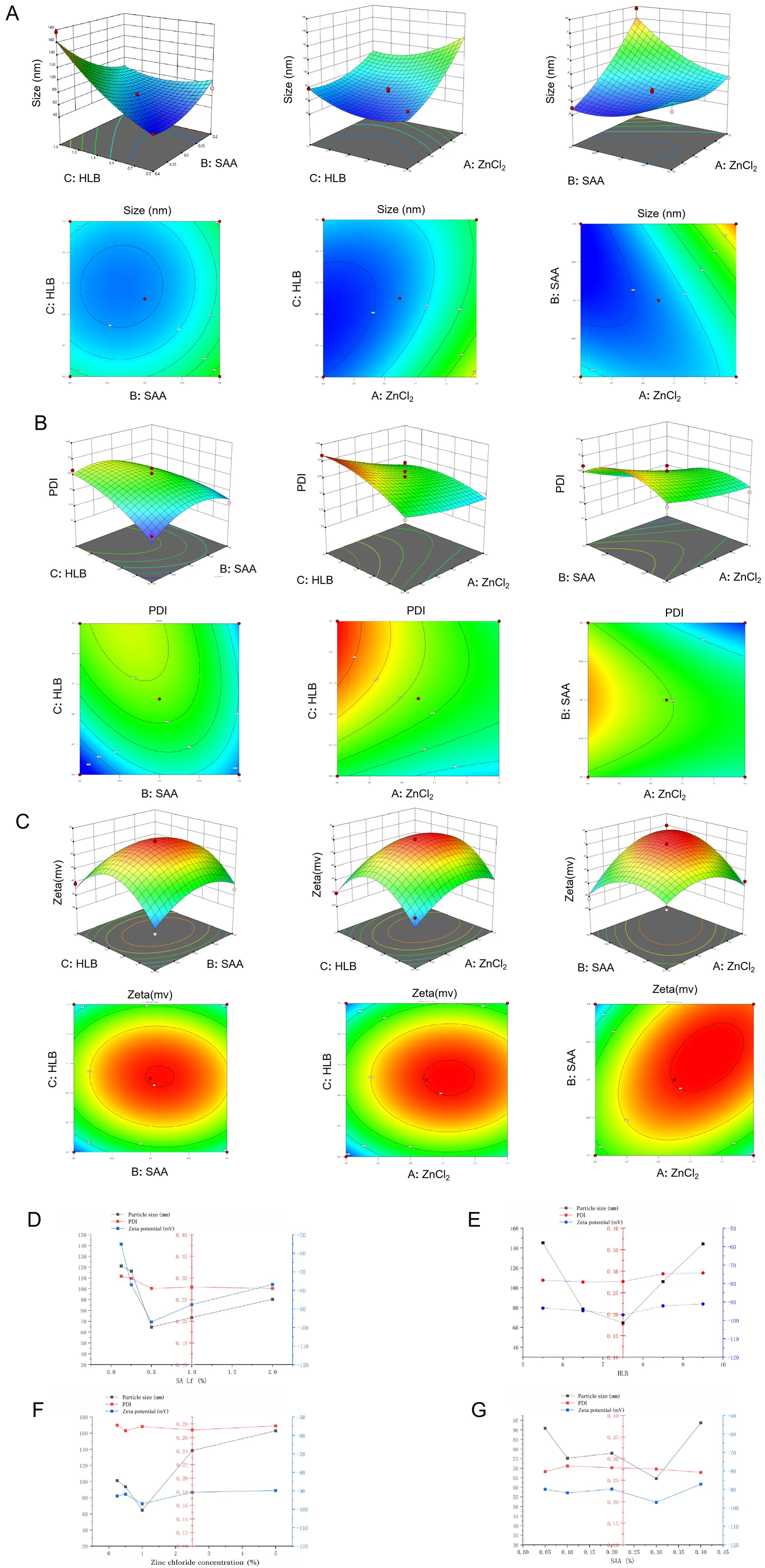
Response surface results. (**A**) Influence on Particle Size. (**B**) Influence on PDI. (**C**) Influence on Zeta Potential. (**D**) Effect of SA-Lf Concentration on Particle Size, PDI, and Zeta Potential. (**E**) Effect of HLB Value on Particle Size, PDI, and Zeta Potential. (**F**) Effect of ZnCl_2_ Concentration on Particle Size, PDI, and Zeta Potential. (**G**) Effect of SAA Concentration on Particle Size, PDI, and Zeta Potential.

The SA-Lf concentration affected the hydrodynamic diameter and zeta potential of the nanogels. At SA-Lf concentrations below 0.50% (*w*/*v*), the particle size increased markedly and reached approximately 110 nm. In comparison, formulations containing 0.50–2.00% SA-Lf showed smaller changes in PDI. The zeta potential also changed progressively with increasing SA-Lf concentration (Figure 2A).

The HLB value primarily affected particle size and particle-size distribution. Within the HLB range of 7.5–9.5, both particle size and PDI tended to increase as the HLB value increased, whereas comparatively smaller changes were observed in zeta potential (Figure 2B).

Variation in ZnCl_2_ concentration resulted in marked changes in the physicochemical properties of the nanogels. Increasing the ZnCl_2_ concentration beyond the intermediate range did not produce a consistent improvement in particle size or PDI, and relatively large particles were observed at some of the higher concentrations. An intermediate ZnCl_2_ concentration was therefore selected for subsequent optimization (Figure 2C).

Increasing the SAA concentration from 0.30% to 0.40% was accompanied by an increase in particle size, whereas the PDI remained below 0.30. Relatively small changes in zeta potential were observed at SAA concentrations of 0.05–0.20%, and the most negative zeta-potential value in the single-factor experiment was observed at an SAA concentration of approximately 0.30% (Figure 2D).

Collectively, the single-factor results supported the use of 0.50% SA-Lf and the further optimization of ZnCl_2_ concentration, HLB value, and SAA concentration by the Box–Behnken design.

#### 2.2.2. Response-Surface Modeling and Optimal Formulation

The complete experimental responses obtained from the 17-run Box–Behnken design are presented in Supplementary Table S2. Across the experimental runs, particle size, PDI, and zeta potential varied substantially with changes in ZnCl_2_ concentration, HLB value, and SAA concentration.

The fitted particle-size model yielded an *R*^2^ value of 0.9070. The overall model was statistically significant (*F* = 6.50, *p* = 0.0168), indicating that the model could adequately describe the particle-size response within the investigated experimental range. Therefore, the particle-size response was included in the subsequent numerical optimization.

The model fitted to PDI was statistically significant (*F* = 8.79, *p* = 0.0078) and explained 92.95% of the observed variability (*R*^2^ = 0.9295). The response-surface plots showed that PDI was influenced by the combined effects of the formulation variables. Lower PDI values were generally observed at HLB values below approximately 7.0, although the magnitude and direction of the response depended on the accompanying ZnCl_2_ and SAA concentrations (Figure 2E).

The zeta-potential model was also statistically significant (*F* = 14.28, *p* = 0.0021) and showed an *R*^2^ value of 0.9554. ZnCl_2_ concentration and its interactions with the other formulation variables contributed substantially to the variation in zeta potential (Figure 2F).

Numerical optimization identified a ZnCl_2_ concentration of 0.972 mg/mL, an HLB value of 6.979, and an SAA concentration of 0.373% (*w*/*v*) as the predicted optimal conditions. Under these conditions, the predicted particle size, PDI, and zeta potential were 80.00 nm, 0.224, and –10.00 mV, respectively.

To validate the predictive reliability of the Box–Behnken optimization model, the optimized formulation was prepared in three independent validation batches under the predicted optimal conditions. The experimentally measured particle size, PDI, and zeta potential were compared with the predicted values, and the prediction errors were calculated. The experimental particle size, PDI, and zeta potential were 82.06 ± 2.16 nm, 0.245 ± 0.005, and –9.54 ± 10.78 mV, respectively. The corresponding prediction errors were 2.57%, 9.38%, and 4.60%, respectively. The raw particle size, PDI, and zeta-potential data from the three independent validation batches are provided in Supplementary Table S3. These results indicate that the Box–Behnken model showed acceptable predictive reliability and could reasonably guide the formulation optimization of SA-Lf-NG@SAA within the investigated design space.

### 2.3. Physicochemical Characteristics of the Optimized SA-Lf-NG@SAA Formulation

Following successful preparation of SA-Lf conjugate, the final SA-Lf-NG@SAA formulation was further characterized by physicochemical properties including particle size, PDI, zeta potential, encapsulation efficiency, drug loading capacity, and in vitro release behavior.

#### 2.3.1. Morphology

**Figure 3:**
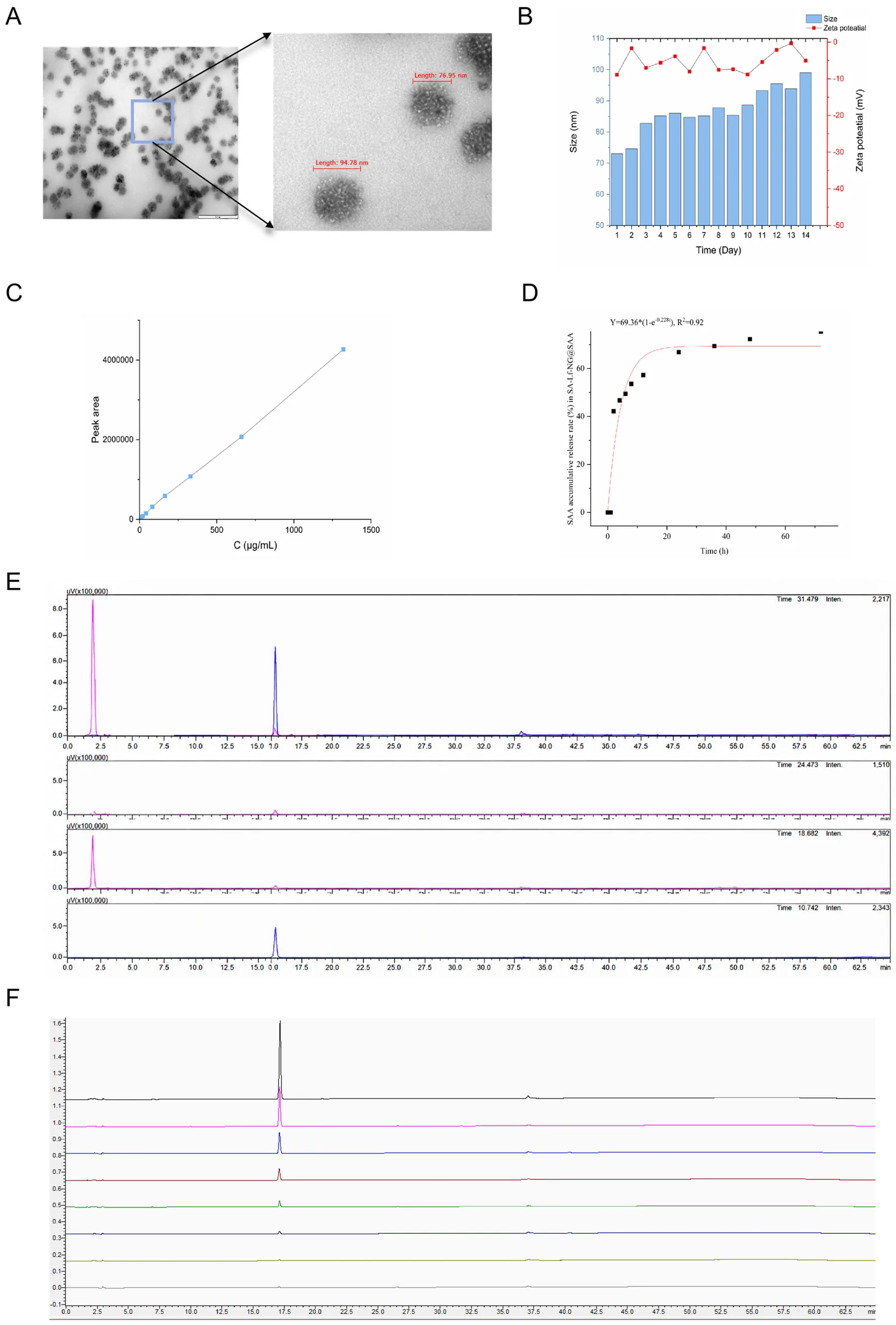
Pharmaceutical Characterization of SA-Lf-NG@SAA Nanogels. (**A**): Appearance of SA-Lf-NG@SAA nanogels. (**B**): Short-term physical stability study of reconstituted SA-Lf-NG@SAA nanogels dispersed in PBS. (**C**): Standard curve under chromatographic conditions. (**D**): In vitro drug release profile of SA-Lf-NG@SAA. (**E**): Method specificity under chromatographic conditions. (**F**): Peak area of the standard curve. In panel (**E**), the red, blue, and black chromatograms represent the sample solution, SAA reference standard, and methanol solvent blank, respectively. In panel (**F**), the chromatograms represent SAA standard solutions at concentrations of 1320, 660, 330, 165, 82.5, 41.25, 20.625, 10.3125, and 5.15625 μg/mL, respectively. The corresponding peak areas were used to construct the calibration curve.

Transmission electron microscopy showed that the optimized SA-Lf-NG@SAA particles were predominantly spherical and displayed a relatively uniform morphological appearance. The particle diameters observed in representative TEM images were approximately 85 nm, although some particle-to-particle variation and limited aggregation were visible in the dried samples (Figure 3A).

#### 2.3.2. Short-Term Physical Stability

During the 14-day short-term physical stability study of the reconstituted SA-Lf-NG@SAA dispersion in PBS at room temperature, the measured particle diameter remained within an approximate range of 75–100 nm. A gradual increase in particle size was observed during the later phase of the study, but no abrupt increase suggestive of extensive aggregation was detected. The zeta potential remained between approximately 0 and –10 mV throughout the observation period (Figure 3B).

These findings indicate that the formulation retained its basic particle characteristics during the 14-day observation period. A gradual increase in particle size was observed during the later phase of storage. This increase may be attributed to gradual hydration and relaxation of the polymeric network, as well as possible rearrangement of the sodium alginate/lactoferrin interface during incubation in PBS. However, no abrupt increase in particle size, visible precipitation, or phase separation was observed, suggesting that the formulation maintained acceptable short-term physical stability. However, this experiment evaluated only the short-term physical stability of the dispersed nanogel system and should not be interpreted as evidence of the long-term storage stability of the final lyophilized formulation.

### 2.4. Analytical Performance of the HPLC Method for SAA

#### 2.4.1. Chromatographic Specificity

Under the established chromatographic conditions (Table 1), the SAA reference standard and SA-Lf-NG@SAA sample produced a distinct chromatographic peak at a retention time of approximately 17.30 min. No evident interfering peak was detected at the SAA retention time in the methanol blank or blank nanogel chromatograms. The SAA peak showed acceptable symmetry without marked tailing (Figure 3E,F).

The gradient program was as follows:

#### 2.4.2. Calibration Linearity

A linear relationship was observed between SAA concentration and chromatographic peak area over the concentration range of 5.15625–1320 μg/mL. The regression equation was:

Y = 3200.64X + 19,446.29(2)

with a coefficient of determination of:

*R*^2^ = 0.9996

indicating satisfactory linearity within the examined concentration range (Figure 3C). The detailed concentration and peak area data used for the construction of the calibration curve are provided in Supplementary Table S4.

#### 2.4.3. Solution Stability and Injection Precision

The peak areas obtained from the same SAA solution at 2, 4, 8, 12, and 24 h showed an RSD of 2.24%, indicating that the test solution remained sufficiently stable during the 24 h analytical period (Supplementary Table S5).

Six consecutive injections of the same test solution yielded an RSD of approximately 2.92% for the SAA concentrations, demonstrating acceptable injection precision under the established chromatographic conditions (Supplementary Table S6).

#### 2.4.4. Accuracy

The accuracy of the HPLC method was evaluated using the recovery test. The recoveries of SAA at low, medium, and high concentration levels were 96.45%, 98.45%, and 104.56%, respectively. The corresponding RSD values were 2.42%, 0.64%, and 0.79%. These results demonstrated good agreement between the measured and theoretical concentrations and indicated satisfactory accuracy of the established HPLC method for SAA determination (Supplementary Table S7).

#### 2.4.5. Precision

The repeatability of the HPLC method was evaluated using six independently prepared SA-Lf-NG@SAA samples. The calculated SAA concentrations ranged from 48.76 to 51.96 μg/mL, with an RSD value of 2.57%, indicating acceptable precision and repeatability of the established analytical method (Supplementary Table S8).

#### 2.4.6. LOD and LOQ

The sensitivity of the HPLC method was further evaluated by determining the LOD and LOQ. Additional low-concentration standard solutions were analyzed, and the calculated LOD and LOQ values were 2.88 μg/mL and 8.74 μg/mL, respectively. These results indicated that the established method possessed sufficient sensitivity for quantitative determination of SAA in SA-Lf-NG@SAA formulations (Supplementary Table S9).

### 2.5. Drug Loading and Encapsulation Efficiency

Three independently analyzed SA-Lf-NG@SAA samples yielded SAA concentrations of 0.1198, 0.0862, and 0.1054 mg/mL after disruption of the nanogel matrix and extraction with methanol.

The mean drug loading of the lyophilized SA-Lf-NG@SAA formulation was:

2.66 ± 0.11%

and the mean encapsulation efficiency was:

75.79 ± 12.31%

The variability in encapsulation efficiency among the three preparations indicated a degree of batch-to-batch variation that should be considered during further process optimization (Supplementary Table S10).

### 2.6. In Vitro Release of SAA from SA-Lf-NG@SAA

Before analyzing the in vitro release profile, the sink condition was assessed based on the measured SAA concentration in PBS and the maximum theoretical SAA concentration in the release medium. To provide a conservative assessment, the lowest measured SAA concentration in PBS, 159.48 μg/mL, was used as the lower limit. In the release study, 35.75, 34.63, and 40.10 mg of SA-Lf-NG@SAA were used in three independent experiments. Based on the measured drug-loading capacity of 2.662%, the maximum theoretical SAA concentrations in 200 mL of release medium were calculated to be 4.76, 4.61, and 5.34 μg/mL, respectively. Even the highest theoretical concentration was far below one-third of the lowest measured SAA concentration in PBS. Therefore, sink conditions were considered to be maintained during the in vitro release study. The raw peak-area data, back-calculated SAA concentrations, and Cmax calculations are provided in Supplementary Table S11.

Minimal detectable release was observed during the initial 0.5–1 h of incubation, indicating an initial lag phase. Thereafter, the cumulative release of SAA increased progressively, reaching approximately 75.44% at 72 h (Figure 3D).

To clarify the release kinetics, the release data were fitted to zero-order, first-order, Higuchi, and Korsmeyer–Peppas models. Because no detectable release was observed during the initial 0–1 h, kinetic fitting of the release phase was performed using the data from 2 to 72 h. As shown in Table 2, the Higuchi model showed the best overall fitting performance among the zero-order, first-order, and Higuchi models, with an R^2^ value of 0.9482 and an RMSE of 2.5950. The Korsmeyer–Peppas model was used to analyze the initial release phase with ($\frac{\mathrm{Mt}}{M\infty }$ < 0.60), and the fitting equation was $\frac{\mathrm{Mt}}{M\infty }$ = 0.3710t^0.1719^, with an R^2^ value of 0.9873. The release exponent n was 0.1719, suggesting that the early-stage release was mainly associated with a diffusion-dominated process. These results indicate that SAA release from SA-Lf-NG@SAA followed a prolonged release pattern after the initial lag phase, with the post-lag release profile being best described by the Higuchi model under the present experimental conditions.

### 2.7. Effects of SA-Lf-NG@SAA on Depression-like Behavior in CUMS Mice

**Figure 4:**
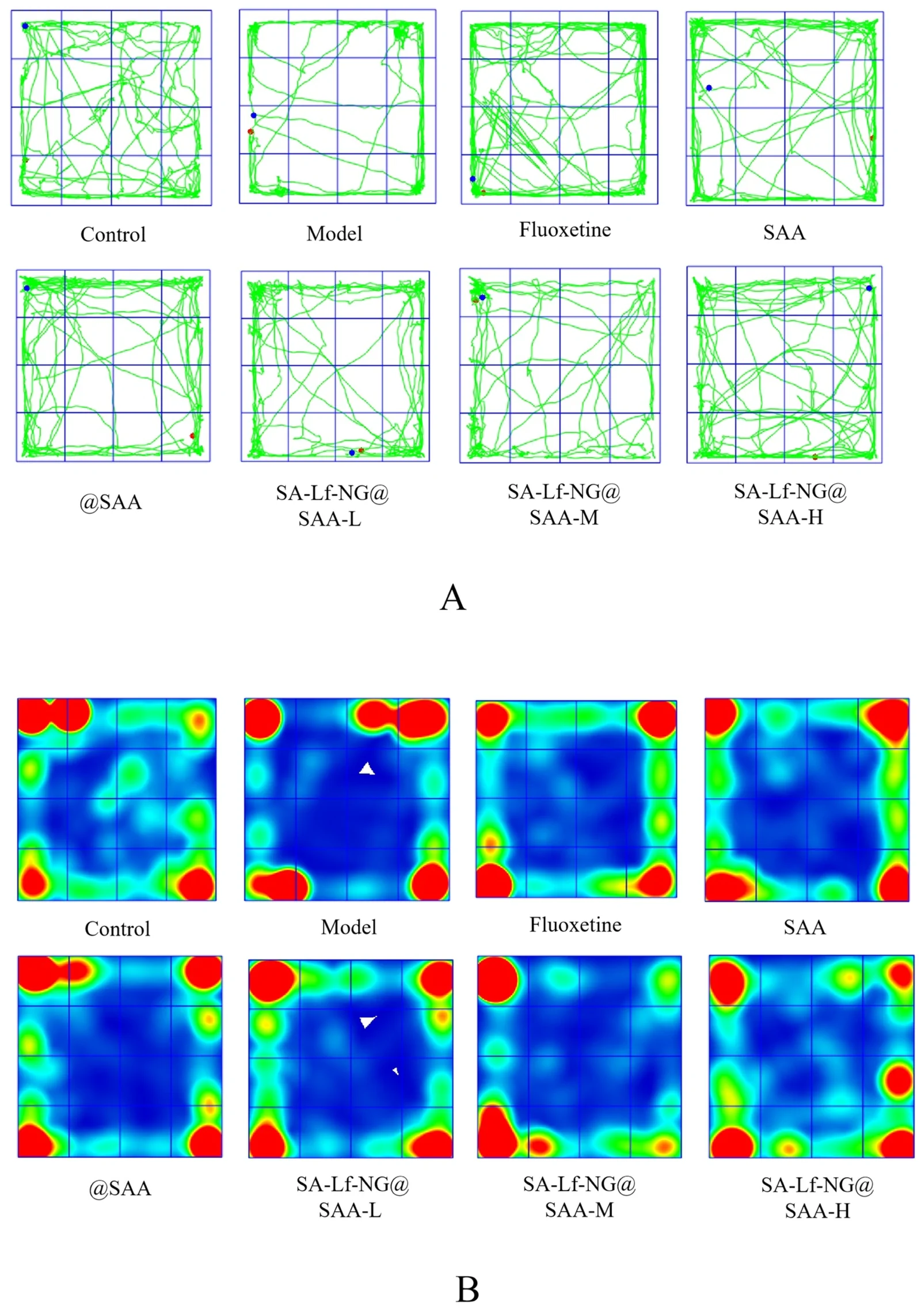
Effects of SA-Lf-NG@SAA treatment on locomotor behavior in CUMS mice assessed by the open field test. (**A**): Trajectory pathways and heat maps of mouse movement. (**B**): Proportional distribution of mouse trajectory areas. Green lines indicate the movement trajectories of the mice. The blue and red dots indicate the starting and ending positions, respectively. In the heat map, colors from blue to red represent increasing residence time/activity intensity. The grid lines divide the open-field arena into equal regions for spatial analysis.

Representative movement trajectories and heat maps showed broad exploratory activity in the Control group, whereas mice in the Model group displayed fewer movement trajectories and reduced spatial exploration. Treatment produced varying degrees of improvement in locomotor activity, with the most evident changes observed in the fluoxetine and high-dose SA-Lf-NG@SAA groups (Figure 4).

#### 2.7.1. Effects on Open-Field Activity

The total distance traveled in the open-field test was significantly lower in the Model group than in the Control group (*p* < 0.001), confirming a marked reduction in spontaneous locomotor and exploratory activity following CUMS exposure.

Compared with the Model group, total distance traveled was significantly increased in the fluoxetine and high-dose SA-Lf-NG@SAA groups (*p* < 0.001). Significant increases were also observed in the oral SAA and medium-dose SA-Lf-NG@SAA groups (*p* < 0.05). The intranasal free-SAA and low-dose SA-Lf-NG@SAA groups showed numerical increases, but the differences did not reach statistical significance in the current analysis (Supplementary Figure S2A).

#### 2.7.2. Effects on Tail-Suspension Immobility

The immobility time in the tail-suspension test was significantly longer in the Model group than in the Control group (*p* < 0.001).

Compared with the Model group, immobility time was significantly reduced by fluoxetine and by the medium- and high-dose SA-Lf-NG@SAA formulations (*p* < 0.001). Oral SAA also reduced immobility time *(p* < 0.01), whereas the low-dose SA-Lf-NG@SAA formulation produced a smaller but statistically significant reduction (*p* < 0.05). The reduction observed after intranasal administration of free SAA did not reach statistical significance (Supplementary Figure S2B).

#### 2.7.3. Effects on Forced-Swimming Immobility

CUMS exposure significantly increased the immobility time in the forced-swimming test compared with the Control group (*p* < 0.001).

Fluoxetine and the medium- and high-dose SA-Lf-NG@SAA formulations significantly reduced immobility time compared with the Model group (*p* < 0.001). Oral SAA also produced a significant reduction (*p* < 0.01). Although lower mean immobility times were observed in the intranasal free-SAA and low-dose SA-Lf-NG@SAA groups, these differences did not reach statistical significance in the current analysis (Supplementary Figure S2C).

Overall, the behavioral findings showed that SA-Lf-NG@SAA produced dose-related antidepressant-like effects, with the medium- and high-dose formulations demonstrating the most consistent effects across the three behavioral tests.

### 2.8. Histopathological Findings in the Striatum and Hippocampus

#### 2.8.1. Striatum

The striatum of Control mice showed relatively intact tissue architecture, evenly distributed cells, and a regular cellular arrangement. In the Model group, the striatal tissue displayed reduced cellular density, disordered cellular arrangement, and an increased number of shrunken cells with abnormal morphology.

**Figure 5:**
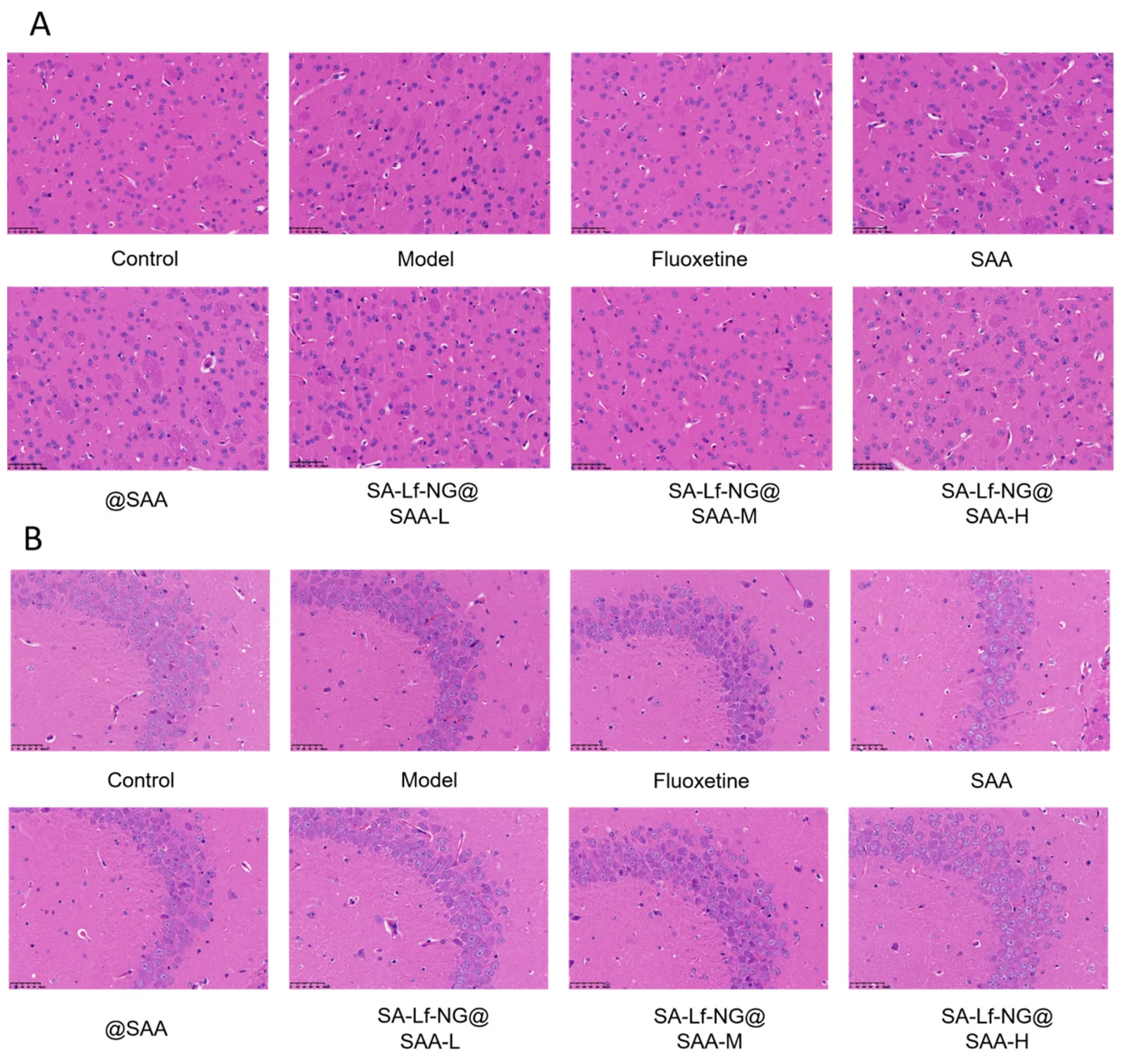
Pathological Sections of Different Brain Regions in Mice (200×). (**A**): HE staining of the striatum in the mouse brain. (**B**): HE staining of the hippocampus in the mouse brain. Scale bar = 50 μm.

Treatment resulted in varying degrees of morphological improvement. The SA-Lf-NG@SAA-treated groups showed more orderly cellular arrangement, greater apparent cellular density, and fewer cells with shrinkage-like changes than the Model group. These differences were most evident in the high-dose SA-Lf-NG@SAA group (Figure 5A).

#### 2.8.2. Hippocampus

The hippocampus of Control mice exhibited a relatively complete structure, distinct nuclear staining, and densely arranged cells. In contrast, the Model group showed loosely arranged cells, reduced cellular density, and more frequent nuclear condensation-like morphological changes.

The treatment groups displayed varying degrees of improvement in hippocampal morphology. In particular, the high-dose SA-Lf-NG@SAA group showed a more compact cellular arrangement, relatively preserved tissue architecture, and fewer nuclear condensation-like changes than the Model group (Figure 5B).

Because the H&E assessment was qualitative, these observations indicate improvement in tissue morphology but do not independently establish neuronal survival or inhibition of apoptosis.

### 2.9. Effects of SA-Lf-NG@SAA on OLFM-Family Protein Expression in the Olfactory Bulb

**Figure 6:**
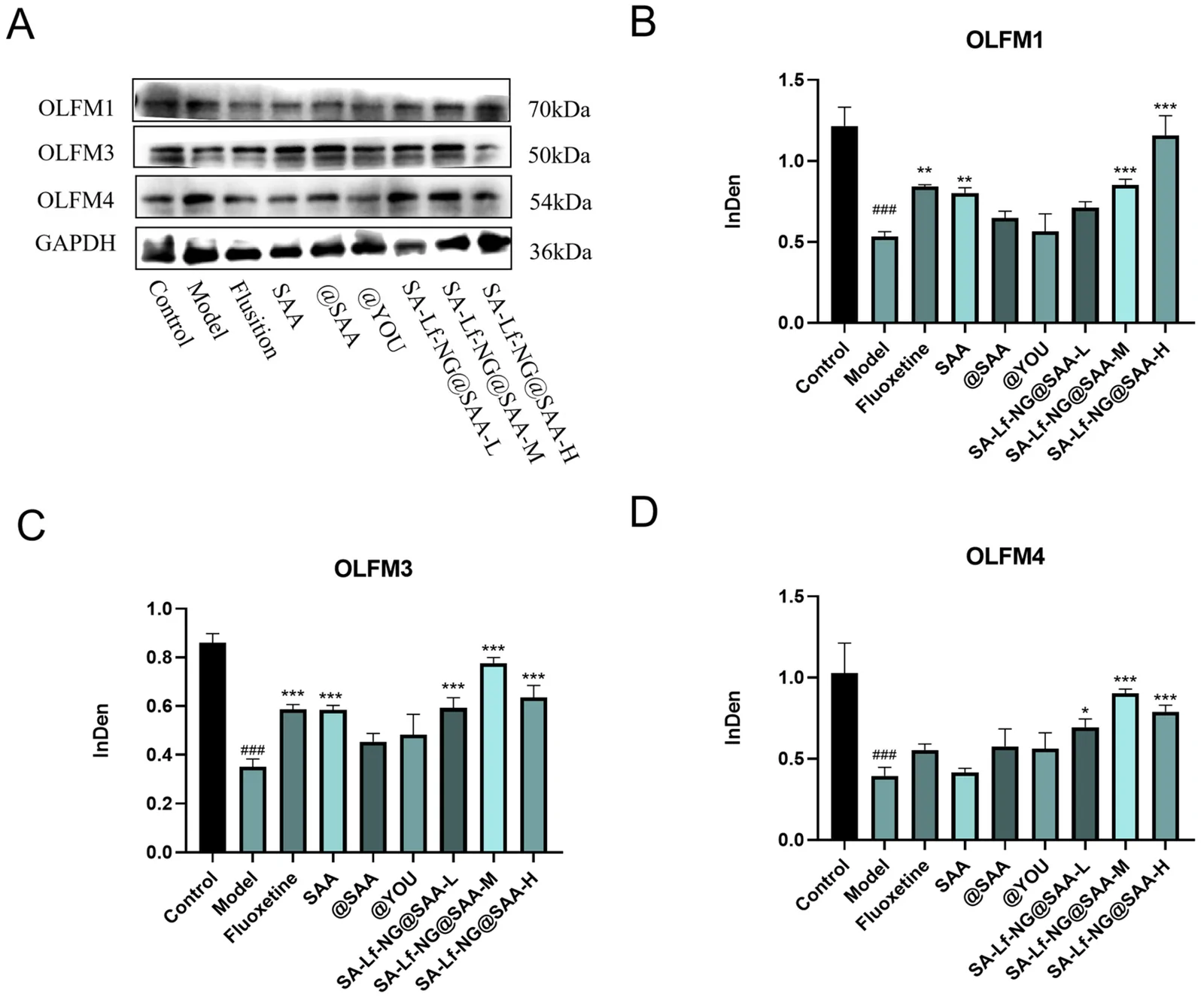
Protein Expression Results in the Mouse Olfactory Bulb. (**A**): Western blot results. (**B**): OLFM-1 expression levels. (**C**): OLFM-3 expression levels. (**D**): OLFM-4 expression levels. Statistical significance was analyzed using one-way ANOVA followed by Dunnett’s multiple-comparisons test, n = 3 biological replicates per group, with the Model group as the reference. ### *p* < 0.001 vs. the Control group; * *p* < 0.05, ** *p* < 0.01, and *** *p* < 0.001 vs. the Model group. Chemiluminescence images were acquired under optimized exposure conditions within the linear detection range. The same acquisition condition was applied for all groups within each target protein. “@YOU” represents an exploratory reference group from a parallel preliminary study and was not included in the statistical comparison or interpretation of SA-Lf-NG@SAA efficacy.

Western blotting showed that OLFM1, OLFM3, and OLFM4 were detectable in the olfactory bulb of Control mice. Compared with the Control group, the expression levels of all three proteins were significantly reduced in the Model group *(p* < 0.001), indicating that CUMS exposure was accompanied by downregulation of OLFM-family proteins in the olfactory bulb (Figure 6A–D).

For OLFM1, fluoxetine and oral SAA significantly increased expression compared with the Model group (*p* < 0.01). The medium- and high-dose SA-Lf-NG@SAA formulations produced more pronounced increases (*p* < 0.001), whereas the increase in the low-dose group did not reach statistical significance.

For OLFM3, significant increases were observed in the fluoxetine, oral SAA, and all three SA-Lf-NG@SAA groups compared with the Model group (*p* < 0.001). Among the nanogel-treated groups, the greatest mean expression was observed in the medium-dose group.

For OLFM4, the low-dose SA-Lf-NG@SAA formulation produced a modest increase compared with the Model group (*p* < 0.05), whereas the medium- and high-dose formulations produced larger increases (*p* < 0.001). The changes observed in the free-SAA groups were less pronounced.

These results show that treatment with SA-Lf-NG@SAA was accompanied by restoration of OLFM-family protein expression in the olfactory bulb, particularly at the medium and high doses.

### 2.10. Effects of SA-Lf-NG@SAA on OLFM-Family Protein Expression in the Hippocampus and Striatum

#### 2.10.1. Hippocampus

Immunofluorescence staining demonstrated detectable OLFM1, OLFM3, and OLFM4 signals in the hippocampus of Control mice. Compared with the Control group, the mean fluorescence intensities of all three proteins were significantly reduced in the Model group (*p* < 0.001).

**Figure 7:**
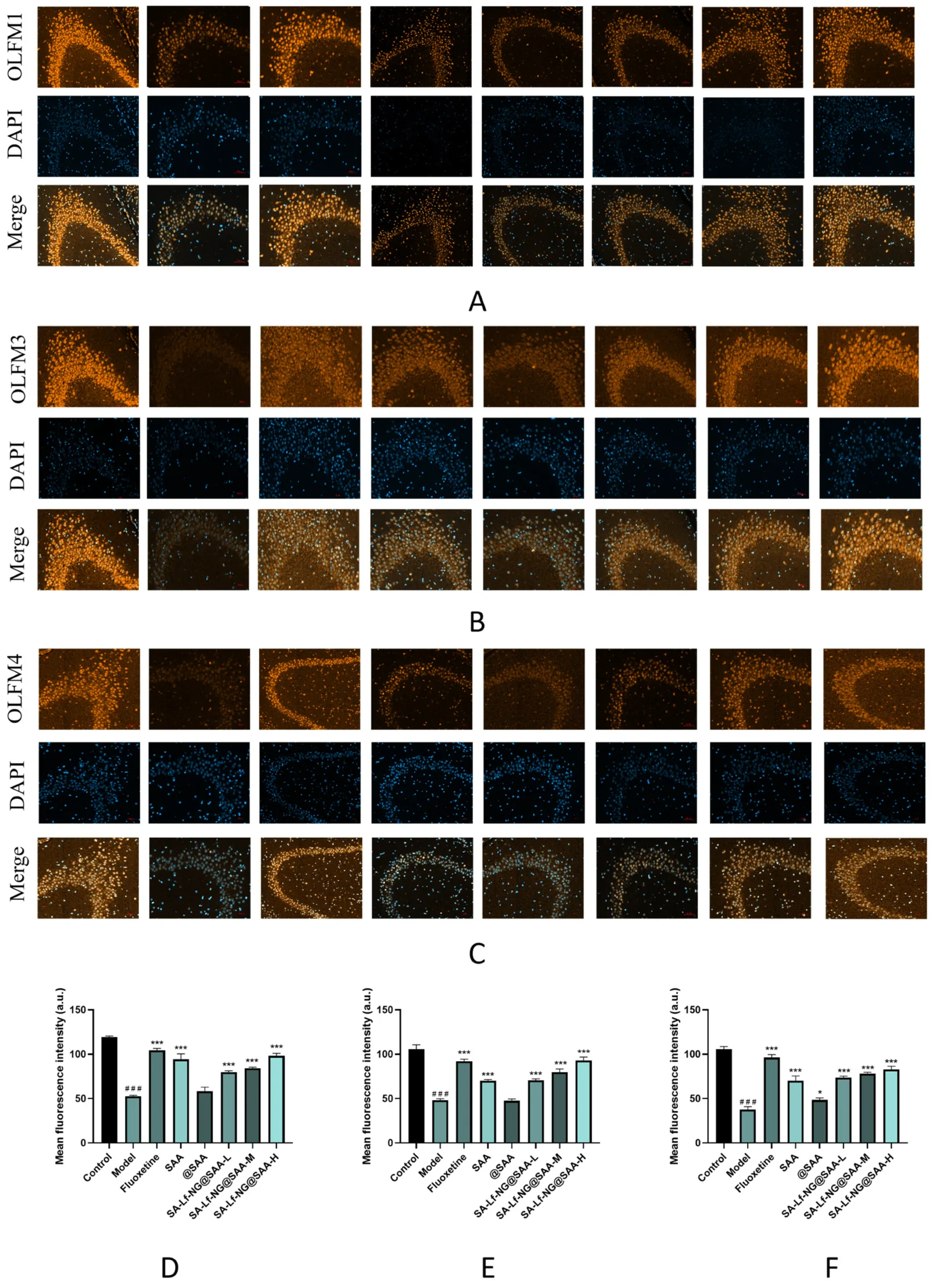
Immunofluorescence detection of OLFM-1, OLFM-3, and OLFM-4 protein expression in the mouse hippocampus. (**A**): Fluorescence expression of OLFM-1 protein. (**B**): Fluorescence expression of OLFM-3 protein. (**C**): Fluorescence expression of OLFM-4 protein. (**D**): Immunofluorescence intensity of OLFM-1 protein. (**E**): Immunofluorescence intensity of OLFM-3 protein. (**F**): Immunofluorescence intensity of OLFM-4 protein. Statistical significance was analyzed using one-way ANOVA followed by Dunnett’s multiple-comparisons test, n = 3 mice per group, with the Model group as the reference. ### *p* < 0.001 vs. the Control group; * *p* < 0.05 and *** *p* < 0.001 vs. the Model group.

Fluoxetine and oral SAA significantly increased hippocampal OLFM1, OLFM3, and OLFM4 fluorescence intensities compared with the Model group. Intranasal free SAA produced smaller changes. All three SA-Lf-NG@SAA groups showed significantly greater fluorescence intensities than the Model group, with the greatest mean values generally observed in the high-dose formulation group (*p* < 0.001; Figure 7A–F).

#### 2.10.2. Striatum

A similar expression pattern was observed in the striatum. OLFM1, OLFM3, and OLFM4 fluorescence intensities were significantly lower in the Model group than in the Control group (*p* < 0.001).

**Figure 8:**
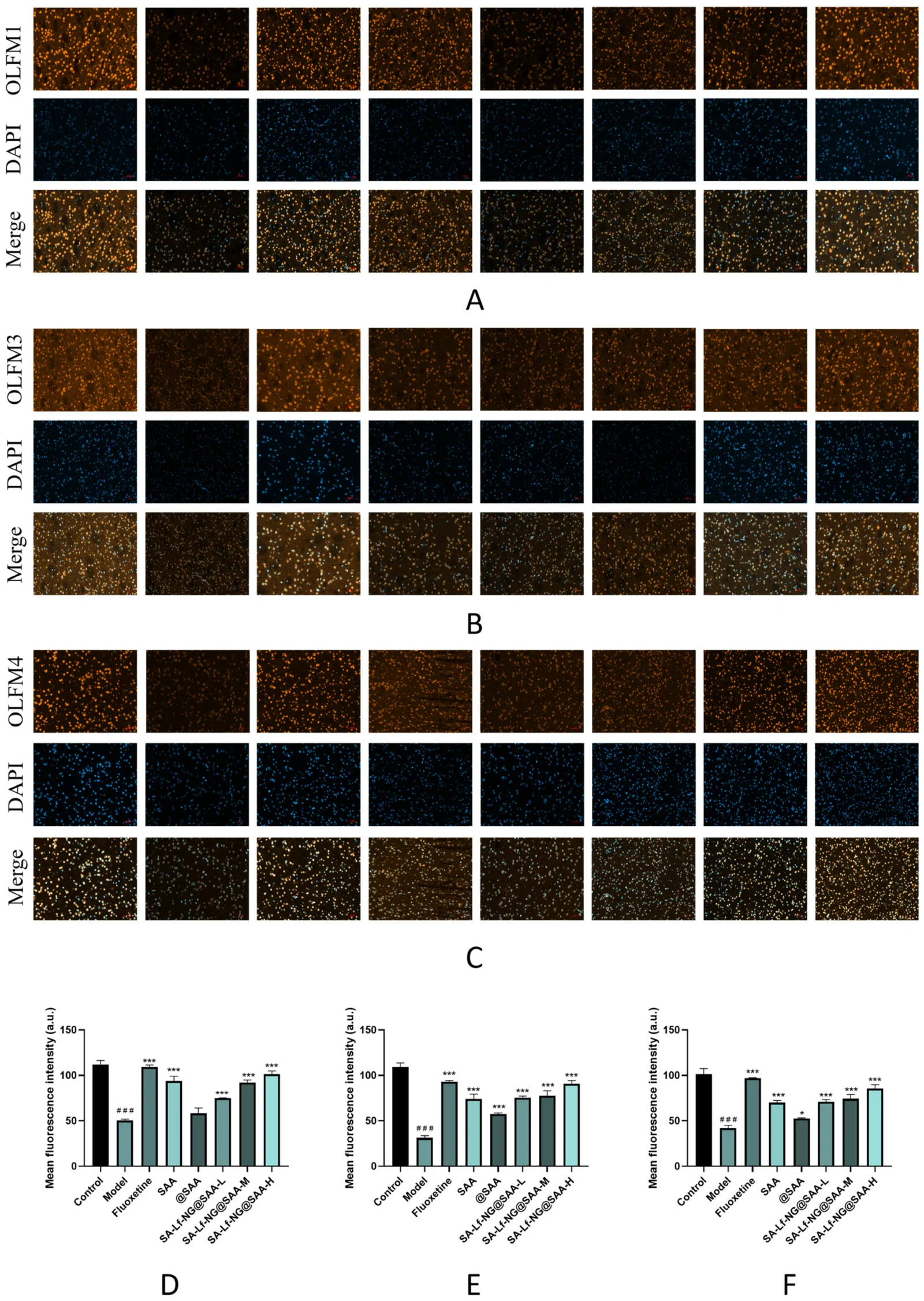
Immunofluorescence detection of OLFM-1, OLFM-3, and OLFM-4 protein expression in the mouse striatum. (**A**): Fluorescence expression of OLFM-1 protein. (**B**): Fluorescence expression of OLFM-3 protein. (**C**): Fluorescence expression of OLFM-4 protein. (**D**): Immunofluorescence intensity of OLFM-1 protein. (**E**): Immunofluorescence intensity of OLFM-3 protein. (**F**): Immunofluorescence intensity of OLFM-4 protein. Statistical significance was analyzed using one-way ANOVA followed by Dunnett’s multiple-comparisons test, n = 3 mice per group, with the Model group as the reference. ### *p* < 0.001 vs. the Control group; * *p* < 0.05 and *** *p* < 0.001 vs. the Model group.

Compared with the Model group, treatment with fluoxetine, oral SAA, and SA-Lf-NG@SAA increased the fluorescence intensities of the three OLFM-family proteins to varying degrees. Significant increases were observed in the low-, medium-, and high-dose SA-Lf-NG@SAA groups, and the high-dose group generally showed the greatest restoration of fluorescence intensity (*p* < 0.001; Figure 8A–F).

Taken together, the Western blot and immunofluorescence results demonstrate that CUMS exposure was associated with reduced OLFM1, OLFM3, and OLFM4 expression in the olfactory bulb, hippocampus, and striatum. SA-Lf-NG@SAA treatment was accompanied by partial restoration of these expression levels, particularly at the medium and high doses. These findings establish an association between the antidepressant-like effects of SA-Lf-NG@SAA and altered OLFM-family protein expression but do not establish a causal role for these proteins.

## 3. Discussion

In the present study, lactoferrin-modified sodium alginate nanogels were developed as an intranasal formulation for the delivery of saikosaponin A. The formulation was optimized and characterized in terms of particle size, particle-size distribution, surface charge, morphology, drug loading, encapsulation efficiency, short-term stability, and in vitro release. Its biological effects were subsequently evaluated in a CUMS mouse model. The principal findings were that SA-Lf-NG@SAA formed nanoscale particles with a relatively uniform morphology, displayed a prolonged in vitro release profile, and produced antidepressant-like behavioral changes accompanied by improved histopathological morphology in the hippocampus and striatum. In addition, treatment was associated with increased expression of OLFM1, OLFM3, and OLFM4 in several brain regions. These findings support the feasibility of SA-Lf-NG@SAA as an intranasal nanogel formulation.

Sodium alginate was selected as the polymeric matrix because of its biocompatibility, abundant carboxyl groups, and ability to undergo ionic crosslinking with divalent metal ions. These properties have enabled its broad application in hydrogel- and nanogel-based drug-delivery systems [15]. In the present formulation, the carboxyl groups of sodium alginate were activated using EDC and NHS and allowed to react with the primary amino groups of lactoferrin. The genipin chromogenic assay, BCA protein determination, and UV-visible spectroscopy consistently indicated the presence of lactoferrin in the SA-Lf product. The estimated lactoferrin grafting ratio was 35.68%, and the appearance of a protein-associated absorption band in the SA-Lf spectrum further supported the incorporation of lactoferrin into the polymeric product.

Single-factor experiments demonstrated that SA-Lf concentration, ZnCl_2_ concentration, surfactant HLB value, and SAA concentration influenced the particle size, PDI, and zeta potential of the nanogels. This is consistent with the expected dependence of ionic-gelation systems on polymer concentration, crosslinking density, interfacial composition, and drug–matrix interactions. An insufficient polymer concentration may produce incomplete or heterogeneous interfacial coverage, whereas excessive polymer or crosslinker concentrations may increase viscosity, promote interparticle bridging, or generate a more compact and heterogeneous crosslinked network. Similarly, changes in the HLB value may affect emulsion formation and the distribution of aqueous droplets within the oil phase, thereby influencing nanogel size and dispersity.

The Box–Behnken design identified a predicted optimal formulation containing approximately 0.972 mg/mL ZnCl_2_, an HLB value of 6.979, and 0.373% SAA. Under the optimized preparation conditions, the obtained nanogels were approximately 85 nm in diameter and were predominantly spherical on TEM examination. Their nanoscale dimensions and relatively uniform morphology may facilitate dispersion within the limited volume available for intranasal administration. The optimized formulation exhibited a weakly negative zeta potential, generally ranging from approximately 0 to −10 mV. Such a low absolute zeta potential suggests that electrostatic repulsion was unlikely to be the principal mechanism maintaining colloidal stability. The short-term consistency observed in the reconstituted PBS dispersion over 14 days may have been partly related to steric stabilization provided by the polymer and surfactants. However, this observation reflects only the short-term physical stability of the dispersed nanogel system and does not provide evidence for the long-term storage stability of the final lyophilized formulation. Further storage stability studies under defined temperature and humidity conditions are required to evaluate the stability of the lyophilized product. The increase in particle size observed during storage indicates that further optimization of crosslinking density and long-term stability evaluation may be required before clinical translation. SA-Lf-NG@SAA had a mean drug loading of approximately 2.66% and an encapsulation efficiency of approximately 75.79%. These findings indicate that most of the initially added SAA was retained in the recovered nanogel fraction.

For intranasal administration, gradual drug release could theoretically prolong exposure at the nasal mucosal surface and reduce rapid drug loss. To clarify the release kinetics, the in vitro release data were fitted to zero-order, first-order, Higuchi, and Korsmeyer–Peppas models. After the initial lag phase, the Higuchi model provided the best overall fit for the 2–72 h release data, suggesting that SAA release from SA-Lf-NG@SAA was mainly associated with diffusion through the hydrated nanogel matrix. The Korsmeyer–Peppas analysis of the initial release phase further yielded a release exponent of 0.1719, supporting a diffusion-dominated early-stage release process. These findings are consistent with the gradual release behavior observed over 72 h. However, the release behavior should be interpreted based on the current in vitro experimental conditions. Although the obtained kinetic analysis provides insights into the prolonged release characteristics of SAA from SA-Lf-NG@SAA, further comparative studies are needed to better understand the detailed release mechanism.

Various intranasal delivery systems, including nanoparticles, liposomal formulations, and hydrogel-based platforms, have been investigated to improve drug stability, prolong residence time, and facilitate therapeutic delivery to the central nervous system. Compared with conventional administration routes, intranasal administration provides a non-invasive approach for administering therapeutic agents through the nasal cavity. In the present study, SA-Lf-NG@SAA demonstrated favorable formulation characteristics for intranasal administration of SAA and showed preliminary antidepressant-like effects in CUMS mice. However, direct comparisons with other delivery systems were not performed, and further studies are required to systematically evaluate the advantages and limitations of different intranasal delivery strategies.

The CUMS model produced behavioral changes consistent with a depression-like phenotype, including reduced activity in the open-field test and increased immobility in the tail-suspension and forced-swimming tests. Treatment with SA-Lf-NG@SAA ameliorated these abnormalities to varying degrees. The reduction in immobility observed in the tail-suspension and forced-swimming tests supports an antidepressant-like effect of SA-Lf-NG@SAA. The open-field findings provide complementary information regarding spontaneous locomotor and exploratory activity. The behavioral effects appeared broadly dose-related, with the medium- and high-dose nanogel formulations producing more consistent responses than the low-dose formulation.

Histological examination showed that CUMS exposure was accompanied by disordered cellular arrangement, reduced apparent cell density, and more frequent shrinkage- or nuclear condensation-like changes in the hippocampus and striatum. These brain regions participate in stress regulation, mood-related behavior, reward processing, cognition, and motor function and are frequently affected in experimental models of chronic stress. SA-Lf-NG@SAA treatment was associated with a more orderly cellular arrangement and fewer apparent morphological abnormalities, particularly at the higher dose. Taken together, the concordant changes in the behavioral tests and histological observations support the preliminary biological activity of the formulation in CUMS mice.

A notable finding of this study was that CUMS exposure was associated with reduced expression of OLFM1, OLFM3, and OLFM4 in the olfactory bulb, hippocampus, and striatum. Treatment with SA-Lf-NG@SAA partially restored the expression of these proteins, with the most pronounced changes generally observed in the medium- and high-dose groups. The regional consistency of the Western blot and immunofluorescence findings suggests that OLFM-family proteins may be responsive to chronic stress and pharmacological intervention.

OLFM1 is expressed in neural tissues and has been implicated in neuronal development, axonal growth, synaptic organization, and neuronal survival [20]. Reduced OLFM1 expression in CUMS mice may therefore reflect alterations in neural plasticity or tissue homeostasis. Its restoration following treatment was temporally associated with behavioral and histological improvement. OLFM3 has been linked to nervous-system development and inflammatory regulation [21]. Because chronic stress can induce neuroimmune and glial responses, the observed changes in OLFM3 may reflect an altered inflammatory or cellular stress state. OLFM4 is more commonly regarded as an immune-associated protein and has been investigated in relation to cellular proliferation, differentiation, inflammatory responses, and apoptosis. It has also been proposed as a potential biomarker associated with major depressive disorder [22]. The increase in OLFM4 expression following SA-Lf-NG@SAA treatment is therefore of exploratory interest.

Nevertheless, several limitations should be acknowledged. First, an Lf-NG group without SAA was not included in the present study, which limits the direct evaluation of the independent contribution of lactoferrin modification to the physicochemical characteristics and biological effects of the nanogel system. Although FTIR analysis provided structural evidence supporting the successful conjugation between SA and Lf, further comparative studies using Lf-NG controls are required to clarify the specific role of lactoferrin modification. Second, an unmodified SA-NG@SAA group was not included in this study, preventing direct evaluation of the specific contribution of lactoferrin modification to the therapeutic efficacy and delivery performance of the formulation. Therefore, the observed effects should be interpreted as the overall performance of the SA-Lf-NG@SAA system rather than being exclusively attributed to lactoferrin modification. In addition, although SA-Lf-NG@SAA was administered intranasally for 14 consecutive days and showed favorable therapeutic effects, dedicated nasal mucosal safety and tolerability evaluations were not performed in the present study. Therefore, the long-term safety of repeated intranasal administration remains to be further investigated. Future studies involving nasal mucosal histopathological analysis, inflammatory response evaluation, and comprehensive safety assessments are required to confirm the clinical applicability of this intranasal delivery system [23,24]. Another limitation of this study is that pharmacokinetic and biodistribution analyses were not performed. Therefore, although SA-Lf-NG@SAA demonstrated improved behavioral outcomes compared with free SAA under the experimental conditions, whether these effects resulted from altered drug exposure remains unclear. Although SA-Lf-NG@SAA showed antidepressant-like effects after intranasal administration, direct evidence of nose-to-brain transport was not obtained in the present study. Further studies using fluorescence imaging, pharmacokinetic analysis, and carrier-control formulations are required to clarify the contribution of lactoferrin modification to intranasal delivery.

## 4. Materials and Methods

The experimental protocol was prepared before the study but was not preregistered.

### 4.1. Materials and Reagents

Sodium alginate (SA; analytical reagent grade, purity ≥ 90%) was purchased from Sigma-Aldrich (Taufkirchen, Germany). Lactoferrin (Lf; purity ≥ 95%), saikosaponin A (SAA; purity ≥ 98%), 1-ethyl-3-(3-dimethylaminopropyl)carbodiimide hydrochloride (EDC), N-hydroxysuccinimide (NHS), genipin (purity ≥ 98%), anhydrous zinc chloride (ZnCl_2_; anhydrous reagent grade, purity ≥ 98%), liquid paraffin (purity ≥ 99%), Span 80, Tween 80, sodium citrate (purity ≥ 99.5%) and 1,1-diphenyl-2-picrylhydrazyl (DPPH; purity ≥ 96%) were purchased from Shanghai Macklin Biochemical Co., Ltd. (Shanghai, China). The Omni-Easy™ ready-to-use BCA protein assay kit was obtained from Shanghai Epizyme Biomedical Technology Co., Ltd. (Shanghai, China). Phosphate-buffered saline (PBS; pH 7.4) was purchased from Zhengzhou Saimosi Biotechnology Co., Ltd. (Zhengzhou, China). HPLC-grade acetonitrile (purity ≥ 99.9%) was purchased from Anhui Tiandi Life Science and Technology Co., Ltd. (Anqing, China), and HPLC-grade methanol was purchased from Tianjin Fuyu Fine Chemical Co., Ltd. (Tianjin, China). Fluoxetine hydrochloride capsules (20 mg/capsule) were obtained from Shanxi Qianyuan Pharmaceutical Group Co., Ltd. (Datong, China).

### 4.2. Synthesis and Characterization of SA-Lf

#### 4.2.1. Synthesis of SA-Lf

SA-Lf was synthesized by EDC/NHS-mediated amidation between the carboxyl groups of SA and the primary amino groups of Lf. Briefly, SA (1.0 g) was dissolved in 100 mL of deionized water and stirred at 40 °C and 600 rpm for 4 h to obtain a 1% (*w*/*v*) SA solution. EDC (958.5 mg, 5 mmol) and NHS (863.2 mg, 7.5 mmol) were added, and the mixture was stirred at 30 °C for 2 h to activate the carboxyl groups of SA [25]. Lf (100 mg) was subsequently added, and the reaction was continued under constant stirring for a total of 24 h [26]. The reaction mixture was transferred to centrifugal ultrafiltration tubes with a molecular-weight cutoff of 100 kDa and centrifuged at 2500 rpm for 30 min. The retained fraction was collected, pre-frozen at −80 °C for 1–2 days, and lyophilized. The resulting SA-Lf conjugate was stored under dry conditions at 4 °C until further use.

#### 4.2.2. Genipin Chromogenic Assay

The presence of Lf in the ultrafiltration fractions was qualitatively evaluated using a genipin chromogenic assay. Genipin was dissolved in ultrapure water and mixed separately with ultrapure water, the ultrafiltration filtrate, the retained fraction, or an Lf solution. The mixtures were vortexed and incubated at room temperature for 12 h. The appearance of a blue color indicated a reaction between genipin and the free amino groups of Lf [27].

#### 4.2.3. Determination of the Lf Grafting Ratio

The Lf content of the SA-Lf conjugate was determined using a BCA protein assay. Lyophilized SA-Lf was dissolved in ultrapure water at a concentration of 1.0 mg/mL. BSA standards were prepared according to the manufacturer’s instructions. Aliquots of 20 μL of each standard or sample were added to a 96-well plate, followed by 200 μL of BCA working reagent. Five replicate wells were used for each sample. After incubation at 37 °C for 30 min, absorbance was measured at 562 nm using a Multiskan™ SkyHigh spectrophotometer (Thermo Scientific, Life Technologies Holdings Pte Ltd., Singapore; made in China). The Lf concentration was calculated from the BSA calibration curve. The Lf grafting ratio was calculated as follows:

Lf grafting ratio (%) = *m*_Lf_/*m*_SA-Lf_ × 100% (3)

where *m*_Lf_ is the mass of Lf detected in the sample and *m*_SA-Lf_ is the total mass of the SA-Lf sample.

#### 4.2.4. UV–Visible Spectroscopy

SA, Lf, and SA-Lf were separately dissolved in ultrapure water. UV-visible spectra were recorded from 200 to 800 nm using a UV-visible spectrophotometer (DU-800, Beckman Coulter, Inc., Brea, CA, USA), with ultrapure water used as the blank.

#### 4.2.5. Fourier-Transform Infrared Spectroscopy

Fourier-transform infrared spectroscopy was performed to further characterize the chemical structures of SA, Lf, and SA-Lf. Dried SA, Lf, and SA-Lf samples were analyzed using a Nicolet iS10 Fourier-transform infrared spectrometer (Thermo Fisher Scientific, Madison, WI, USA). The spectra were recorded at room temperature over the range of 4000–500 cm^−1^. The characteristic absorption bands of SA, Lf, and SA-Lf were compared to evaluate the structural changes associated with EDC/NHS-mediated conjugation.

### 4.3. Preparation of SA-Lf-NG@SAA

SA-Lf was dispersed in ultrapure water at the required concentration and stirred at 40 °C and 600 rpm for 4 h. The solution was subsequently stored at 4 °C for 6 h to allow complete hydration. Span 80 and Tween 80 were mixed in proportions corresponding to the required hydrophilic–lipophilic balance value. For an HLB value of approximately 7.5, 3.5 mL of Span 80 and 1.5 mL of Tween 80 were mixed and stirred at room temperature for 3 h. The HLB value was calculated as follows:

(4) $$\mathrm{HLB}=\frac{\mathrm{HL}B_{\mathrm{Span}}\times V_{\mathrm{Span}}+\mathrm{HL}B_{\mathrm{Tween}}\times V_{\mathrm{Tween}}}{V_{\mathrm{Span}}+V_{\mathrm{Tween}}}$$

Anhydrous ZnCl_2_ (300 mg) was dissolved in 30 mL of 70% ethanol to prepare a 10 mg/mL stock solution, which was diluted to the required concentration before use. SAA was incorporated into the ZnCl_2_ solution before crosslinking. Liquid paraffin (50 mL) and mixed surfactant (0.5 mL) were stirred at 40 °C and 500 rpm for 30 min. SA-Lf solution (15 mL) was then added at 0.75 mL/min using a peristaltic pump (BT100-3J, Baoding Longer Precision Pump Co., Ltd., Baoding, China) while the stirring speed was increased to 1000 rpm. After complete addition, emulsification was continued for 1 h. The SAA-containing ZnCl_2_ solution (5 mL) was subsequently added at 0.15 mL/min, followed by stirring for an additional 1 h to induce ionic crosslinking. The resulting suspension was centrifuged at 2000 rpm for 15 min, and the upper oil phase was removed. The nanogel-containing fraction was collected, pre-frozen at −80 °C, and lyophilized. The resulting SA-Lf-NG@SAA powder was stored under dry, refrigerated conditions. Blank SA-Lf nanogels were prepared using the same procedure without the addition of SAA.

### 4.4. Optimization of SA-Lf-NG@SAA by Single-Factor Experiments and Box–Behnken Design

#### 4.4.1. Single-Factor Experiments

The effects of SA-Lf concentration, ZnCl_2_ concentration, HLB value, and SAA concentration on particle size, polydispersity index (PDI), and zeta potential were evaluated using single-factor experiments. When one factor was varied, the remaining preparation conditions were kept constant. The evaluated levels were as follows:

SA-Lf concentration: 0.125%, 0.25%, 0.50%, 1.0%, and 2.0% (*w*/*v*);

ZnCl_2_ concentration: 0.25, 0.50, 1.0, 2.5, and 5.0 mg/mL;

HLB value: 5.5, 6.5, 7.5, 8.5, and 9.5;

SAA concentration: 0.05%, 0.10%, 0.20%, 0.30%, and 0.40% (*w*/*v*).

During the investigation of SAA concentration, the SA-Lf concentration, ZnCl_2_ concentration, and HLB value were maintained at 0.50% (*w*/*v*), 1.0 mg/mL, and 7.5, respectively. The detailed experimental design is provided in Supplementary Tables S12–S15.

#### 4.4.2. Box–Behnken Design

Based on the single-factor results, ZnCl_2_ concentration, HLB value, and SAA concentration were selected as independent variables for optimization using a three-factor, three-level Box–Behnken design (Table 3) [28,29]. The SA-Lf concentration was fixed at 0.50% (*w*/*v*).

A total of 17 experimental runs, including five center-point replicates, were performed. Particle size, PDI, and zeta potential were used as response variables. Experimental design, regression analysis, and generation of response-surface plots were performed using Design-Expert version 13.0.1.0 (Stat-Ease, Inc., Minneapolis, MN, USA).

The experimental data were fitted to a second-order polynomial model:

(5) $$Y=\beta_{0}+\sum_{i=1}^{3}\beta_{i}X_{i}+\sum_{i=1}^{3}\beta_{\mathrm{ii}}X_{i}^{2}+\sum_{i<j}\beta_{\mathrm{ij}}X_{i}X_{j}$$

where Y is the predicted response; β_0_ is the intercept; β_i_, β_ii_, and β_ij_ are the linear, quadratic, and interaction coefficients, respectively; and X_i_ and X_j_ are the coded independent variables. Numerical optimization was performed to obtain an appropriate particle size, a low PDI, and an acceptable zeta potential. The predicted optimal formulation was subsequently prepared and characterized experimentally.

To validate the reliability of the optimized formulation predicted by the Box–Behnken model, the optimized SA-Lf-NG@SAA formulation was prepared in three independent validation batches according to the predicted optimal conditions. Particle size, PDI, and zeta potential were measured as described above. The prediction error was calculated using the following equation:

(6) $$\mathrm{Prediction}\;\mathrm{error}\;(\%)=\frac{\left|\mathrm{Experimental}\;\mathrm{value}-\mathrm{Predicted}\;\mathrm{value}\right|}{\left|\mathrm{Predicted}\;\mathrm{value}\right|}\times 100\%$$

### 4.5. Particle Size, PDI, Zeta Potential, Stability, and Morphology

The hydrodynamic diameter and PDI of SA-Lf-NG@SAA were measured by dynamic light scattering, and the zeta potential was determined by electrophoretic light scattering using a particle-size and zeta-potential analyzer (90Plus, Brookhaven Instruments Corporation, Nashua, NH, USA). Samples were dispersed in ultrapure water and diluted 20-fold before measurement. Measurements were performed at 25 °C using three independently prepared samples.

For the short-term physical stability study of the reconstituted nanogel dispersion, lyophilized SA-Lf-NG@SAA was dispersed in PBS at pH 7.4 and maintained at room temperature. Particle size, PDI, and zeta potential were measured once daily for 14 consecutive days. Visible aggregation, precipitation, and phase separation were also recorded. This experiment was designed to evaluate the short-term physical stability of SA-Lf-NG@SAA after dispersion in PBS and was not intended to assess the long-term storage stability of the final lyophilized formulation.

For transmission electron microscopy, an appropriately diluted nanogel dispersion was placed onto a copper grid and air-dried at room temperature. Morphology was examined using a transmission electron microscope (JEM-1400, JEOL Ltd., Tokyo, Japan) operated at an acceleration voltage of 200 kV.

### 4.6. HPLC Analysis of SAA

#### 4.6.1. Chromatographic Conditions

SAA was quantified using an HPLC system (CBM-20A, Shimadzu Corporation, Kyoto, Japan) equipped with a UV detector and a ZORBAX Eclipse XDB-C18 column (250 mm × 4.6 mm, 5 μm; Agilent Technologies, Santa Clara, CA, USA). Acetonitrile and water were used as mobile phases A and B, respectively.

#### 4.6.2. Specificity

Specificity was evaluated by comparing chromatograms of a methanol blank, an SAA reference-standard solution, a blank nanogel solution, and an SA-Lf-NG@SAA sample solution. The absence of interfering peaks at the retention time of SAA was used to assess method specificity.

#### 4.6.3. Linearity

An SAA stock solution was prepared in methanol and serially diluted to concentrations of 5.15625, 10.3125, 20.625, 41.25, 82.5, 165, 330, 660, and 1320 μg/mL. The calibration curve was constructed by plotting SAA peak area against concentration. Linear regression was used to obtain the calibration equation and coefficient of determination.

#### 4.6.4. Injection Precision

Injection precision was evaluated by analyzing the same sample solution six consecutive times. The SAA peak areas were recorded, and the relative standard deviation was calculated.

#### 4.6.5. Solution Stability

The stability of the SAA sample solution was evaluated by repeated analysis at 2, 4, 8, 12, and 24 h after preparation. Stability was assessed from the RSD of the SAA peak areas.

#### 4.6.6. Accuracy Evaluation

The accuracy of the HPLC method was evaluated by a recovery experiment. Blank SA-Lf-NG samples were spiked with known amounts of SAA standard solution at three concentration levels (low, medium, and high). The spiked samples were subsequently treated using the same sample preparation procedure as that used for SAA quantification. The recovery (%) was calculated according to the following equation:

(7) $$\mathrm{Recovery}\;(\%)=\frac{\mathrm{Measured}\;\mathrm{concentration}}{\mathrm{Theoretical}\;\mathrm{concentration}}\times 100\%$$

The results were expressed as mean recovery and relative standard deviation (RSD).

#### 4.6.7. Precision Evaluation

The precision of the HPLC method was assessed by repeatability testing. Six independently prepared SA-Lf-NG@SAA samples were obtained from the same batch of formulation. Each sample was accurately weighed and processed following the established sample preparation procedure. The samples were analyzed under identical chromatographic conditions, and the RSD value of the calculated SAA concentrations was determined to evaluate the repeatability of the analytical method.

#### 4.6.8. LOD and LOQ Determination

The sensitivity of the HPLC method was evaluated by determining the limit of detection (LOD) and limit of quantification (LOQ). Additional low-concentration SAA standard solutions were analyzed for sensitivity evaluation. The LOD and LOQ values were calculated according to the ICH guidelines based on the standard deviation of the response and the slope of the calibration curve using the following equations:

(8) $$LOD=\frac{3.3\sigma }{S}$$

(9) $$\mathrm{LOQ}=\frac{10\sigma }{S}$$

where σ represents the standard deviation of the response and S represents the slope of the calibration curve.

### 4.7. Drug Loading and Encapsulation Efficiency

Three independently prepared SA-Lf-NG@SAA samples were analyzed. Lyophilized nanogels were accurately weighed and dispersed in 3 mL of sodium citrate solution to disrupt the crosslinked gel network. Methanol (7 mL) was added, and the samples were sonicated for 30 min.

After centrifugation at 2000 rpm for 20 min, the supernatant was collected and filtered through a 0.45 μm membrane. The SAA concentration was determined using the HPLC method described above [30].

Drug loading was calculated as follows:

(10) $$\mathrm{DLC}\;(\%)=\frac{\mathrm{mass}\;\mathrm{of}\;\mathrm{SAA}\;\mathrm{in}\;\mathrm{the}\;\mathrm{recovered}\;\mathrm{nanogels}}{\mathrm{mass}\;\mathrm{of}\;\mathrm{the}\;\mathrm{recovered}\;\mathrm{nanogels}}\times 100$$

Encapsulation efficiency was calculated as follows:

(11) $$\mathrm{EE}\;(\%)=\frac{\mathrm{mass}\;\mathrm{of}\;\mathrm{SAA}\;\mathrm{in}\;\mathrm{the}\;\mathrm{recovered}\;\mathrm{nanogels}}{\mathrm{initial}\;\mathrm{mass}\;\mathrm{of}\;\mathrm{SAA}\;\mathrm{added}}\times 100$$

Results were expressed as the mean ± standard deviation of three independently prepared samples.

### 4.8. In Vitro Release

A predetermined amount of SA-Lf-NG@SAA was dispersed in 2 mL of PBS at pH 7.4 and transferred into a pretreated dialysis bag (Shanghaiyuanye Bio-Technology Co., Ltd., Shanghai, China) with a molecular-weight cutoff of 8–14 kDa. The dialysis bag was immersed in 200 mL of PBS maintained at 37 °C under continuous agitation at 500 rpm.

At 0.5, 1, 2, 4, 6, 8, 12, 24, 36, 48, and 72 h, 1 mL of release medium was withdrawn and immediately replaced with an equal volume of fresh PBS maintained at 37 °C [31,32]. The SAA concentration in each sample was determined by HPLC.

The cumulative amount released at the nth sampling time was calculated as follows:

(12) $$Q_{n}=C_{n}V_{0}+V_{s}\sum_{i=1}^{n-1}C_{i}$$

The cumulative release percentage was calculated as follows:

(13) $$\mathrm{Cumulative}\;\mathrm{release}\;(\%)=\frac{Q_{n}}{Q_{\mathrm{total}}}\times 100$$

where *C*_n_ is the SAA concentration at the nth sampling time, V_0_ is the total volume of release medium, *V*_s_ is the sampling volume, and Q_total_ is the total amount of SAA in the nanogel sample.

The in vitro release data were fitted to commonly used kinetic models, including zero-order, first-order, Higuchi, and Korsmeyer–Peppas models. Because no detectable SAA release was observed during the initial 0–1 h, this period was considered an initial lag phase, and the kinetic fitting of the release phase was performed using the data from 2 to 72 h. The equations were as follows:

(14) $$\mathrm{zero}\text{-}\mathrm{order}\;\mathrm{model},\;Q_{t}=k_{0}t+C$$

(15) $$\mathrm{first}\text{-}\mathrm{order}\;\mathrm{model},\;ln(100-Q_{t})=ln100-k_{1}t$$

(16) $$\mathrm{Higuchi}\;\mathrm{model},\;Q_{t}/Q_{\infty }=k_{H}t^{1/2}+C$$

(17) $$\mathrm{Korsmeyer}–\mathrm{Peppas}\;\mathrm{model},\;M_{t}/M_{\infty }=kt^{n}$$

where Q_t_ represents the cumulative release percentage at time t; k_0_, k_1_, k_H_, and k are the release rate constants of the corresponding models; and n is the release exponent. For the Korsmeyer–Peppas model, only the initial release data with $M_{t}/M_{\infty }<0.60$ were used for fitting. The goodness of fit was evaluated using R^2^ and RMSE. Kinetic fitting was performed using Origin software version 9.9.0.225 (OriginLab Corporation, Northampton, MA, USA).

The solubility of SAA in PBS at pH 7.4 was evaluated to determine whether sink conditions were maintained during the in vitro release study. Briefly, SAA was added to PBS at pH 7.4 and incubated at 37 °C under continuous shaking. After equilibration, the samples were centrifuged, and the supernatant was collected and filtered through a 0.45 μm membrane. The SAA concentration in the supernatant was determined by HPLC. The maximum theoretical SAA concentration in the release medium was calculated based on the amount of SA-Lf-NG@SAA used in the release experiment, the measured drug-loading capacity, and the total volume of the release medium. Sink conditions were considered to be maintained when the maximum theoretical SAA concentration was lower than one-third of the measured SAA solubility in PBS.

The release profile was fitted using nonlinear regression in Origin software version 9.9.0.225 (OriginLab Corporation, Northampton, MA, USA). All experiments were performed in triplicate.

The DPPH radical scavenging assay was performed as an additional in vitro evaluation, and the detailed procedure and results are provided in the Supplementary Materials.

### 4.9. Pharmacodynamics Study

#### 4.9.1. Animals

Ninety-six specific-pathogen-free male C57BL/6J mice, aged 7–8 weeks and weighing 18–22 g, were obtained from Shandong Jinan Pengyue Laboratory Animal Co., Ltd. (Jinan, China; license no. SCXK [Lu] 20220006; animal certificate No. 370726241101442342). The mice were housed in the Experimental Animal Center of Henan University of Chinese Medicine under controlled environmental conditions of 24 °C, 45%, and a 12-h light/12-h dark cycle, with free access to food and water except during scheduled stress procedures. The sample size was determined based on previous studies using CUMS models, preliminary experiments, and the principle of minimizing animal use while ensuring sufficient statistical power. Mice were randomly allocated to experimental groups using a computer-generated randomization sequence. To minimize potential confounders, behavioral testing was conducted by investigators blinded to group allocation, and animals from different groups were tested under the same behavioral sequence to minimize experimental bias.

#### 4.9.2. CUMS Procedure

Twelve mice were assigned to the normal-control group and maintained under standard housing conditions. The remaining mice were exposed to chronic unpredictable mild stress for 4 weeks. The stressors included 24-h food deprivation, 24-h water deprivation, cold-water swimming at 10 °C for 5 min, warm-water swimming at 40 °C for 5 min, cage tilting at 45°, damp bedding, and reversal of the light/dark cycle. One or two stressors were applied each day according to an unpredictable schedule, and consecutive repetition of the same stressor was avoided (Supplementary Table S17) [33]. Animals were monitored daily for body weight, general appearance, food and water intake, and abnormal behaviors. After swimming stress, mice were dried and kept warm until recovery. Humane endpoints included severe weight loss, persistent inability to eat or drink, marked lethargy, or severe distress. No unexpected adverse events occurred during the experiment.

#### 4.9.3. Grouping and Treatment

After the CUMS modeling period, the stressed mice were randomly allocated to seven groups, with 12 mice per group: Model group, Fluoxetine group, Oral SAA group, Intranasal SAA group, Low-dose SA-Lf-NG@SAA group, Medium-dose SA-Lf-NG@SAA group, High-dose SA-Lf-NG@SAA group. Together with the normal-control group, eight experimental groups were included. Fluoxetine was selected as a positive control because it is a clinically approved antidepressant widely used for the treatment of depression. In the present study, fluoxetine treatment was included to provide a reference for evaluating the antidepressant-like effects of SA-Lf-NG@SAA in the CUMS model. The purpose of this comparison was not to demonstrate superiority over fluoxetine, but rather to assess the therapeutic response relative to an established antidepressant treatment.

Mice in the normal-control and model groups received 8 μL of normal saline by intranasal administration. Mice in the fluoxetine group received fluoxetine hydrochloride by oral gavage at 12.5 mg/kg. Mice in the oral SAA group received free SAA by oral gavage at 10 mg/kg. Mice in the intranasal SAA group received free SAA intranasally at 2 mg/kg. Based on the measured drug loading of 2.662%, SA-Lf-NG@SAA suspensions were prepared at concentrations of 192, 384, and 576 mg/mL. A total intranasal volume of 8 μL was administered to each mouse, corresponding to SAA doses of approximately 2, 4, and 6 mg/kg in the low-, medium-, and high-dose groups, respectively [34,35]. All treatments were administered once daily for 14 consecutive days. Behavioral tests were conducted after treatment, followed by tissue collection. The experimental unit was an individual mouse. The mice not exposed to CUMS were used as the normal control group.

### 4.10. Behavioral Tests

Mice were transferred to the behavioral-testing room at least 30 min before each test. The behavioral tests were conducted on separate days in the following order: open-field test (OFT), tail-suspension test (TST), and forced-swimming test (FST), with a 24-h interval between consecutive tests. The OFT was conducted first to assess spontaneous locomotor and exploratory activity before exposure to the more stressful behavioral procedures. The TST and FST were subsequently performed as complementary assessments of depression-like behavior, with a 24-h interval between tests to minimize potential carry-over effects. The apparatus was cleaned with 70% ethanol between animals to minimize residual olfactory cues. The primary behavioral outcome measures included total distance traveled and the percentage of movement distance in the central zone in the open-field test, as well as immobility time in the tail suspension test and forced swimming test. Representative movement trajectories were generated to visualize the locomotor and exploratory patterns of mice in each group.

#### 4.10.1. Open-Field Test (OFT)

Each mouse was placed in the center of an open-field arena measuring 0.25 m^2^. Locomotor and exploratory activity were recorded for 5 min using a video-tracking system (XR-XZ301, Shanghai Xinruan Information Technology Co., Ltd., Shanghai, China). The total distance traveled and the proportion of movement distance in each predefined region were analyzed [36].

#### 4.10.2. Tail-Suspension Test (TST)

Each mouse was suspended by the distal one-third of the tail using adhesive tape. The test lasted 5 min, and the cumulative immobility time was recorded. Immobility was defined as the absence of initiated body movements, excluding movements caused by respiration or passive swinging [37].

#### 4.10.3. Forced-Swimming Test (FST)

Each mouse was placed individually in a transparent cylinder containing water maintained at 23–25 °C. The test lasted 5 min. Immobility was defined as the absence of active escape-directed behavior, with only minimal movements necessary to keep the head above water. Total immobility time was recorded [38].

### 4.11. Histological Examination

Following the behavioral tests, mice designated for histological examination were deeply anesthetized with sodium pentobarbital (50 mg/kg, intraperitoneally). Retro-orbital blood collection was performed under deep anesthesia by trained personnel. The mice were then euthanized by cervical dislocation, and death was confirmed before tissue collection. Brain tissues containing the hippocampus and striatum were collected and fixed in 4% paraformaldehyde for 24–48 h.

The tissues were dehydrated through graded ethanol, cleared in xylene, embedded in paraffin, and sectioned at a thickness of 4–5 μm. Sections were deparaffinized, rehydrated, and stained with hematoxylin and eosin.

Histological morphology of the hippocampus and striatum was examined using a light microscope (Axioscan 7, Carl Zeiss Microscopy GmbH, Jena, Germany) at 200× magnification. Tissue integrity, neuronal arrangement, cell density, and nuclear condensation-like morphological changes were evaluated. Histopathological changes in the hippocampus and striatum were assessed as secondary outcomes.

### 4.12. Western Blotting and Immunofluorescence

The secondary molecular outcome measures included the expression levels of OLFM-family proteins, including OLFM-1, OLFM-3, and OLFM-4. Western blot analysis was used to quantify the expression of OLFM-1, OLFM-3, and OLFM-4 in the olfactory bulb, with GAPDH used as the internal reference. Immunofluorescence staining was performed to evaluate the spatial expression and fluorescence intensity of OLFM-1, OLFM-3, and OLFM-4 in depression-related brain regions, including the hippocampus and striatum.

#### 4.12.1. Western Blotting

Olfactory-bulb tissues were collected and stored at −80 °C until analysis. Total protein was extracted using lysis buffer containing protease inhibitors and quantified using the BCA method.

Approximately 16 μg of protein per sample was separated by 10% SDS-PAGE and transferred to PVDF membranes. The membranes were blocked with 5% skim milk at room temperature for 40 min and incubated at 4 °C for approximately 10 h with primary antibodies against OLFM1 (DF6064##58; 1:1000; Affinity, Shanghai, China), OLFM3 (bs-11061R; 1:1000; Bioss, Beijing, China), OLFM4 (bsm-61792R; 1:1000; Bioss, China), and GAPDH (LF211; 1:5000; Epizyme, Shanghai, China).

After washing, the membranes were incubated with HRP-conjugated goat anti-rabbit IgG (RGAR001; 1:5000; Proteintech, Rosemont, IL, USA) at room temperature for 30 min. Protein bands were visualized using enhanced chemiluminescence and quantified using ImageJ software version 1.54f (National Institutes of Health, Bethesda, MD, USA). OLFM1, OLFM3, and OLFM4 expression levels were normalized to GAPDH.

#### 4.12.2. Immunofluorescence Staining

Paraffin-embedded brain sections containing the hippocampus and striatum were deparaffinized and rehydrated. Antigen retrieval was performed using citrate buffer at pH 6.0. After cooling and washing, the sections were blocked with blocking solution at room temperature for 30 min.

Sections were incubated overnight at 4 °C with primary antibodies against OLFM1, OLFM3, or OLFM4. After washing, the sections were incubated with the corresponding fluorescence-conjugated secondary antibodies for 1 h at room temperature in the dark. Nuclei were counterstained with DAPI, and the sections were mounted using an antifade mounting medium.

Images were acquired from anatomically matched regions using a fluorescence microscope (Axioscan 7, Carl Zeiss Microscopy GmbH, Jena, Germany) under identical acquisition settings. Three mice per group were analyzed, and one anatomically matched field per section was captured. Mean fluorescence intensity was quantified using ImageJ after background subtraction. The mean value obtained from each animal was used as the statistical unit.

### 4.13. Statistical Analysis

Statistical analyses were performed using GraphPad Prism software version 8.2.1 (GraphPad Software, San Diego, CA, USA) and Design-Expert software where appropriate. Quantitative data are presented as mean ± standard deviation (SD) or mean ± standard error of the mean (SEM), as indicated.

For formulation optimization, the Box–Behnken design data were analyzed using response surface methodology. A second-order polynomial regression model was established, and analysis of variance (ANOVA) was performed to evaluate the significance and adequacy of the fitted model.

For HPLC method validation, calibration curves were generated using linear regression analysis. Injection precision and solution stability were evaluated by calculating the relative standard deviation (RSD). Drug loading and encapsulation efficiency were expressed as mean ± SD from independent measurements.

The in vitro release profiles were analyzed by nonlinear regression fitting.

For animal experiments, quantitative behavioral data, including open-field test, tail-suspension test, and forced-swimming test results, were analyzed using one-way analysis of variance (one-way ANOVA), followed by Dunnett’s multiple-comparisons test. Western blot densitometric data and immunofluorescence intensity data for OLFM1, OLFM3, and OLFM4 were analyzed separately using one-way ANOVA followed by Dunnett’s multiple-comparisons test. The Model group was used as the reference group for multiple comparisons. The comparison between the Control and Model groups was used to evaluate the effect of CUMS induction, whereas comparisons between treatment groups and the Model group were used to evaluate therapeutic effects.

Histopathological observations of the hippocampus and striatum were evaluated qualitatively and were not subjected to inferential statistical analysis.

All statistical tests were two-sided, and *p* < 0.05 was considered statistically significant.

## 5. Conclusions

In this study, lactoferrin-modified sodium alginate nanogels loaded with saikosaponin A were successfully developed as an intranasal formulation. The optimized SA-Lf-NG@SAA formulation exhibited nanoscale dimensions, a relatively uniform spherical morphology, moderate drug loading, and satisfactory encapsulation efficiency. It also showed a gradual and prolonged SAA release profile over 72 h under the present in vitro conditions.

In CUMS mice, intranasal administration of SA-Lf-NG@SAA produced dose-related antidepressant-like effects, as indicated by improved performance in the open-field, tail-suspension, and forced-swimming tests. Treatment was also associated with attenuation of stress-related histopathological alterations in the hippocampus and striatum. Furthermore, SA-Lf-NG@SAA partially restored OLFM1, OLFM3, and OLFM4 expression in the olfactory bulb, hippocampus, and striatum. These protein changes should be regarded as exploratory molecular correlates of the observed behavioral and histological effects rather than evidence of a causal OLFM-mediated mechanism.

Overall, the findings support the preliminary feasibility of SA-Lf-NG@SAA as a formulation for intranasal administration of SAA and provide a basis for further investigation. However, because an unmodified SA-NG@SAA group was not included, the present study cannot determine whether lactoferrin modification provided additional advantages over unmodified sodium alginate nanogels. Therefore, the antidepressant-like effects observed in this study should be interpreted as effects of the SA-Lf-NG@SAA formulation as a whole, rather than being specifically attributed to lactoferrin modification. Direct comparisons with blank and non-lactoferrin-modified nanogels, together with nasal safety, pharmacokinetic, biodistribution, and functional mechanistic studies, are required in future work.

## Figures and Tables

**Table 1:** Mobile Phase Conditions.

Time/Min	Mobile Phase A (%)	Mobile Phase B (%)
0–50	25 → 90	75 → 10
50–55	90	10

Note: ”→” indicates a linear gradient from the initial percentage to the final percentage over the specified time interval. The flow rate was 1.0 mL/min, the column temperature was maintained at 35 °C, the injection volume was 10 μL, and the detection wavelength was 210 nm.

**Table 2:** Kinetic fitting results of SAA release from SA-Lf-NG@SAA.

Model	Data Range	Fitting Equation	R^2^	RMSE
Zero-order	2–72 h	Q_t_ = 0.4592t + 48.4313	0.8345	4.6390
First-order	2–72 h	ln(100 − Q_t_) = 3.9543 − 0.01224t	0.8989	3.6892
Higuchi	2–72 h	Q_t_ = 4.8290t^1/2^ + 38.6071	0.9482	2.5950
Korsmeyer–Peppas	2–12 h	$\frac{Mt}{M\infty }$ = 0.3710t^0.1719^	0.9873	0.5963

Q_t_ represents the cumulative release percentage at time t. For the first-order model, the R^2^ value was calculated from the linear regression of ln(100 − Q_t_) versus t, whereas RMSE was calculated based on the back-calculated cumulative release percentage. For the Korsmeyer–Peppas model, only the initial release data with $\frac{\mathrm{Mt}}{M\infty }$ < 0.60 were used for fitting.

**Table 3:** Experimental Design for Response Surface Methodology (RSM).

Coded Level	a-ZnCl_2_ Concentration (mg/mL)	b-HLB Value	c-SAA Concentration, % (*w*/*v*)
−1	0.5	6.5	0.2
0	1.0	7.0	0.3
1	1.5	7.5	0.4

## Data Availability

The original contributions presented in this study are included in the article/Supplementary Material. Further inquiries can be directed to the corresponding authors.

## Supplementary Materials

The following supporting information can be downloaded at https://www.mdpi.com/article/10.3390/ph19091425/s1, Figure S1: Lf grafting ratio; Figure S2: Quantitative analysis of Behavior test parameters in CUMS mice after different treatments; Figure S3: Antioxidant activity of SA-Lf-NG@SAA; Table S1: Lactoferrin Grafting Ratio Results; Table S2 Results of the Response Surface Experiment; Table S3: Raw validation data of the optimized SA-Lf-NG@SAA formulation; Table S4: Relationship between SAA Concentration and Peak Area; Table S5: Table Peak Area of Reference Standard Solution Within 24 Hours; Table S6: Injection precision; Table S7: Accuracy evaluation of the HPLC method for SAA determination by recovery test; Table S8: Repeatability evaluation of the HPLC method for SAA determination; Table S9: Sensitivity evaluation of the HPLC method for SAA determination; Table S10: Drug Loading Capacity and Entrapment Efficiency of SA-Lf-NG@SAA; Table S11: Assessment of sink conditions for the in vitro release study; Table S12: Experimental Design for the Single-Factor Study of SA-Lf Concentration; Table S13: Experimental Design for the Single-Factor Study of ZnCl_2_ Concentration; Table S14: Experimental Design for the Single-Factor Study of HLB Value; Table S15: Experimental Design for the Single-Factor Study of SAA Concentration; Table S16: Composition of Sample Solutions; Table S17: CUMS stressor schedule. Method and Result S1: In vitro DPPH radical scavenging assay.

## Abbreviations

SA, sodium alginate; Lf, lactoferrin; CUMS, chronic unpredictable mild stress; OLFM-1, olfactomedin 1; OLFM-3, olfactomedin 3; OLFM-4, olfactomedin 4; SAA, Saikosaponin A; EDC, N- (3-dimethylaminopropyl)-N’-ethyl-carbodiimide; NHS, N-Hydroxysuccinimide; HLB, Hydrophilic Lipophilic Balance; PDI, Polymer Dispersity Index; DPPH, 1,1-diphenyl-2-picryl-hydrazyl radical; FST, Forced Swim Test; TST, Tail Suspension Test; OFT, Open field test.
