# Supplementary material for: Antidepressant Effects of Lactoferrin-Modified Sodium Alginate-Based Saikosaponin A Nanogels via Intranasal Administration: Involvement of Olfactomedin-Family Proteins
Source: Pharmaceuticals (Basel). 2026 Sep 9;19(9):1425. doi: 10.3390/ph19091425 (PMC13610083; doi:10.3390/ph19091425)
Supplement: Supplementary file 1 [file pharmaceuticals-19-01425-s001.zip › pharmaceuticals-4504099-supplementary.pdf]

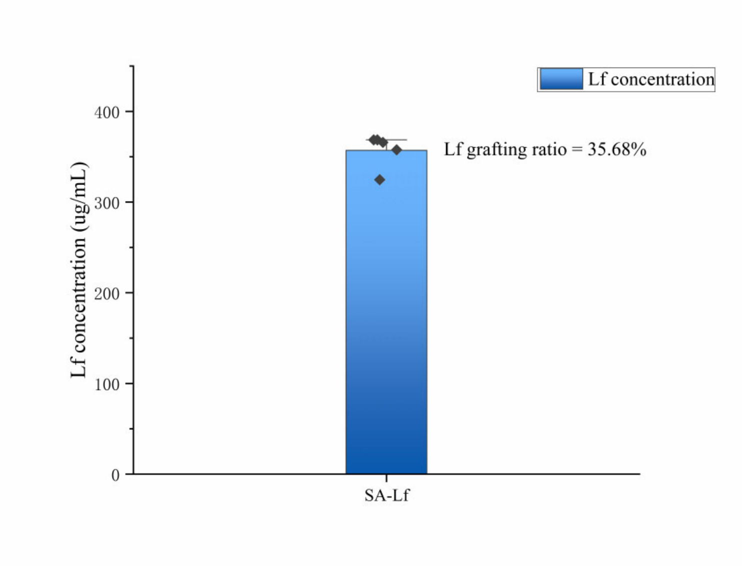

Supplementary Figure S1. Lf grafting ratio.

Supplementary Table S1: Lactoferrin Grafting Ratio Results

Lf（ug/ml）	average	Lf Grafting Ratio
367.255		
365.881		
324.673	356.815±8.259	35.68%
357.640		
368.628		

Supplementary Table S2: Results of the Response Surface Experiment

NO.	a-$ZnCl_2$（mg/mL）	b-HLB	c-SAA（m/v，%）	Particle Size（nm）	PDI	Zeta Potential（mV）
1	0.5	7	0.2	73.3533	0.232667	-53.9333
2	0.5	7	0.4	50.7266	0.271	-83.61
3	1	6.5	0.4	101.52	0.162	-70.2433
4	1	7	0.3	70.8651	0.2423	0.38
5	1.5	6.5	0.3	106.36	0.299	-77.01
6	1	7	0.3	69.1021	0.25014	1.12
7	1	7	0.3	65.213	0.24165	1.53
8	0.5	7.5	0.3	78.23	0.318333	-79.5267
9	1.5	7.5	0.3	92.2833	0.227333	-66.5867
10	1	7.5	0.4	99.6767	0.164333	-57.3967

11	1.5	7	0.2	82.4133	0.188333	-55.8133
12	1	6.5	0.2	96.21	0.154	-88.27
13	1	7	0.3	67.7967	0.255667	0.35
14	0.5	6.5	0.3	72.44	0.218	-68.81
15	1	7.5	0.2	99.1	0.265333	-61.9133
16	1.5	7	0.4	176.5	0.171	0.16
17	1	7	0.3	63.75	0.27167	-0.86

Supplementary Table S3: Raw validation data of the optimized SA-Lf-NG@SAA formulation

Batch	Particle size (nm)	PDI	Zeta potential (mV)
1	84.32	0.240	-21.83
2	81.83	0.245	-1.68
3	80.02	0.250	-5.11
Mean	82.06 ± 2.16	0.245 ± 0.005	−9.54 ± 10.78

Supplementary Table S4: Relationship between SAA Concentration and Peak Area

Number	1	2	3	4	5	6	7	8	9
Peak area	4268330	2070685	1075466	588620	311562	149633	78198	45373	20335
C（μg/ml）	1320	660	330	165	82.5	41.25	20.625	10.3125	5.15625

Supplementary Table S5: Table Peak Area of Reference Standard Solution Within 24 Hours

Time	2	4	8	12	24
peak areas	608019	616534	622130	643855	632666
RSD/%			2.24		

Supplementary Table S6: injection precision

Ingredient	Peak Area	c（mg/ml）	RSD/%
	231340	0.06617	
	232410	0.06653	
SAA	236730	0.06789	2.92
	248690	0.07162	
	234545	0.06722	
	239396	0.06871	

Supplementary Table S7: Accuracy evaluation of the HPLC method for SAA determination by recovery test

Added level	Theoretical concentration (μg/ml)	Measured concentration (μg/ml)	Recovery (%)	RSD (%)
Low	72.33	69.76±1.69	96.45	2.42
Medium	97.95	96.46±0.61	98.45	0.64
High	113.77	118.91±0.94	104.56	0.79

Data are expressed as mean ± SD (n = 3).

Supplementary Table S8: Repeatability evaluation of the HPLC method for SAA determination

Sample NO.	Peak area	Calculated SAA concentration (μg/ml)
1	175523	48.76
2	185761	54.96
3	185055	51.74
4	178246	49.61
5	181100	50.51
6	184708	51.63
Mean	50.70±1.31	
RSD	2.57%	

Supplementary Table S9: Sensitivity evaluation of the HPLC method for SAA determination

Parameter	Result
Concentrtion range used for sensitivity evaluation	0.322 - 82.5 μg/ml
Regression equation	Y= 3746.76X + 557.75
Correlation coefficient (R^2)	0.9991
LOD	2.88 μg/ml

LOQ	8.74 μg/ml

Supplementary Table S10: Drug Loading Capacity and Entrapment Efficiency of SA-Lf-NG@SAA

NO.	SAA（mg/ml）	DLC/%	EE/%
L1	0.1198		
L2	0.0862	2.662±0.1105	75.7933±12.31
L3	0.1054		

Data are presented as mean ± SD from three independent measurements.

Supplementary Table S11: Assessment of sink conditions for the in vitro release study

A. Back-calculated SAA concentration in PBS

Sample	Peak area	Dilution factor	Calculated SAA concentration (μg/mL)
0.5mg/mL	2383554	1	738.63
0.25mg/mL	1103569	1	338.71
0.125mg/mL	529917	1	159.48
Lowest measured concentration used foe conservative assessment	-	-	159.48
One-third of the lowest measured concentration	-	-	53.16

B. Maximum theoretical SAA concentration in the release medium

Replicate	Nanogel mass used in release study(mg)	Theoretical SAA amount based on DLC (μg)	Release medium volume (mL)	Maximum theortical SAA concentration Cmax (μg/mL)
1	35.75	951.67	200	4.76
2	34.63	921.85	200	4.61
3	40.10	1067.46	200	5.34
Mean ± SD	36.83 ± 2.89	980.33±76.92	-	4.90 ± 0.38
Highest Cmax	-	-	-	5.34
Sink condition judgment	-	-	-	Maintained

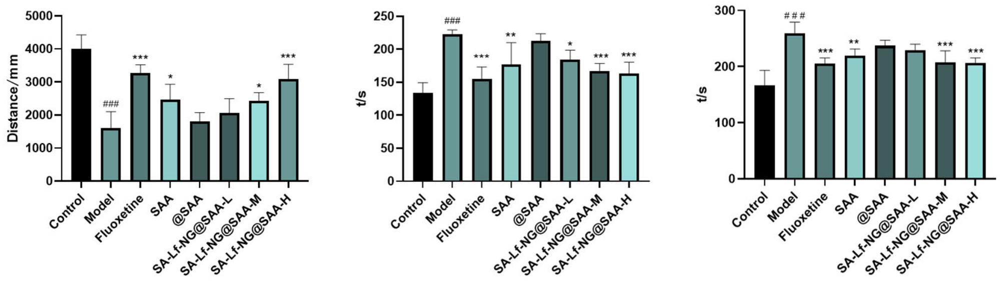

A B C

Supplementary Figure S2. Quantitative analysis of Behavior test parameters in CUMS mice after different treatments.(**A**): Total distance traveled by mice in the Open Field Test. (**B**): Immobility time of mice in the tail suspension test. (**C**): Immobility time of mice in the forced swimming test. Statistical significance was analyzed using one-way ANOVA followed by Dunnett's multiple-comparisons test, with the Model group as the reference, n = 6 mice per group. ###*P < 0.001* vs. the Control group; **P < 0.05*, ***P < 0.01*, and ****P < 0.001* vs. the Model group.

Supplementary Table S12: Experimental Design for the Single-Factor Study of SA-Lf Concentration

SA-Lf (m/v,%)	$ZnCl_2$ (mg/mL)	HLB	SAA (m/v, %)
0.125	1	7.5	0.3
0.25	1	7.5	0.3
0.5	1	7.5	0.3
1	1	7.5	0.3
2	1	7.5	0.3

Supplementary Table S13: Experimental Design for the Single-Factor Study of $ZnCl_2$ Concentration

SA-Lf (m/v,%)	$ZnCl_2$ (mg/mL)	HLB	SAA (m/v, %)
0.5	0.25	7.5	0.3
0.5	0.5	7.5	0.3
0.5	1	7.5	0.3
0.5	2.5	7.5	0.3
0.5	5	7.5	0.3

Supplementary Table S14: Experimental Design for the Single-Factor Study of HLB Value

SA-Lf	$ZnCl_2$	HLB	SAA

(m/v,%)	(mg/mL)		(m/v, %)
0.5	1	5.5	0.3
0.5	1	6.5	0.3
0.5	1	7.5	0.3
0.5	1	8.5	0.3
0.5	1	9.5	0.3

Supplementary Table S15: Experimental Design for the Single-Factor Study of SAA Concentration

SA-Lf (m/v,%)	$ZnCl_2$ (mg/mL)	HLB	SAA (m/v, %)
0.5	1	7.5	0.05
0.5	1	7.5	0.10
0.5	1	7.5	0.2
0.5	1	7.5	0.3
0.5	1	7.5	0.4

Supplementary Method and Result S1: In vitro DPPH radical scavenging assay

Twenty milligrams each of SA-Lf-NG@SAA and blank SA-Lf-NG were separat ely dispersed in 1 mL of purified water. An appropriate amount of SAA stand ard was dissolved in absolute ethanol to prepare the SAA standard solution. A DPPH stock solution was prepared by dissolving 2.5 mg of DPPH in 50 mL of absolute ethanol and was further diluted fivefold to obtain a 10 μg/mL wo rking solution.

The reaction systems were prepared according to Supplementary table S5. Each group was prepared in triplicate. After incubation, the absorbance was measur ed at 517 nm at 0.5, 1, 2, and 4 h. The DPPH radical scavenging activity wa s calculated according to the following equation:

$$\text{DPPH radical scavenging rate} = \frac{C0+B-A}{C0}\times 100\% \quad (8)$$

As shown in Supplementary Figure S1, SA-Lf-NG@SAA exhibited DPPH radic al scavenging activity in vitro, whereas blank SA-Lf-NG showed relatively wea k scavenging activity. The scavenging activity of SA-Lf-NG@SAA increased wi th incubation time, suggesting that the SAA-loaded nanogels retained certain an

tioxidant activity in vitro.

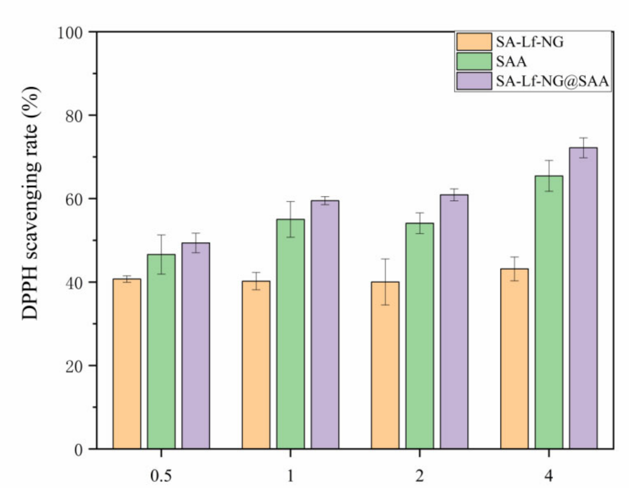

Supplementary Figure S3. Antioxidant activity of SA-Lf-NG@SAA

Supplementary Table S16: Composition of Sample Solutions

	Composition
A1	25 μL SA-Lf-NG@SAA+975 μL DPPH
A2	25 μL SA-Lf-NG+975 μL DPPH
A3	25 μL SAA+975 μL DPPH
B1	25 μL SA-Lf-NG@SAA+975 μL Absolute Ethanol
B2	25 μL SA-Lf-NG+975 μL Absolute Ethanol
B3	25 μL SAA+975 μL Absolute Ethanol
C0	25 μL Absolute Ethanol+975 μL DPPH

Supplementary Table S17: CUMS stressor schedule

Day	Stressor 1	Stressor 2
Day 1	Cage tilt at 45°	Cold-water swimming
Day 2	Food deprivation	Stroboscopic light stimulation
Day 3	Hot-water swimming	Reversed light/dark cycle
Day 4	Mild cage shaking	Water deprivation
Day 5	Cage tilt at 45°	Mild cage tapping/disturbance
Day 6	Cold-water swimming	Mild cage shaking
Day 7	Stroboscopic light stimulation	Hot-water swimming
Day 8	Reversed light/dark cycle	Food deprivation
Day 9	Water deprivation	Mild cage tapping/disturbance
Day 10	Cage tilt at 45°	Food deprivation
Day 11	Cold-water swimming	Reversed light/dark cycle

Day 12	Hot-water swimming	Mild cage shaking
Day 13	Stroboscopic light stimulation	Water deprivation
Day 14	Cage tilt at 45°	Mild cage tapping/disturbance
Day 15	Cold-water swimming	Reversed light/dark cycle
Day 16	Stroboscopic light stimulation	Hot-water swimming
Day 17	Food deprivation	Mild cage shaking
Day 18	Water deprivation	Mild cage tapping/disturbance
Day 19	Cage tilt at 45°	Cold-water swimming
Day 20	Food deprivation	Stroboscopic light stimulation
Day 21	Hot-water swimming	Reversed light/dark cycle
Day 22	Mild cage shaking	Water deprivation
Day 23	Cage tilt at 45°	Mild cage tapping/disturbance
Day 24	Cold-water swimming	Reversed light/dark cycle
Day 25	Food deprivation	Mild cage shaking
Day 26	Stroboscopic light stimulation	Water deprivation
Day 27	Cage tilt at 45°	Hot-water swimming
Day 28	Cold-water swimming	Mild cage tapping/disturbance
